# Subsoil Material Heterogeneity in a Multi-Span Viaduct: Identification and Operational Deflection Shape Assessment of Wave Propagation and Application to Train-Induced Vibrations

**DOI:** 10.3390/ma19214730

**Published:** 2026-11-06

**Authors:** Paweł Boroń, Joanna Maria Dulińska, Dorota Jasińska

**Affiliations:** Faculty of Civil Engineering and Transport, PK Krakow University of Technology, 31-155 Kraków, Poland; joanna.dulinska@pk.edu.pl (J.M.D.); dorota.jasinska@pk.edu.pl (D.J.)

**Keywords:** subsoil heterogeneity, road-substructure materials, wave propagation, vibration attenuation, spatially variable kinematic excitation, Operational Deflection Shape, train-induced vibrations

## Abstract

This study investigates the influence of subsoil material heterogeneity on the dynamic performance of a five-span road viaduct with spans crossing different ground zones, including native subsoil and compacted, mechanically stabilized road-substructure materials. A spatially varying kinematic excitation model based on the wave-passage approach was adopted to represent the spatial variation in support excitation. Controlled vibroseis tests with synchronized acceleration measurements at viaduct supports were used to experimentally identify section-specific apparent propagation velocities by cross-correlation and material absorption coefficients by least-squares fitting of the amplitude-decay relationship. Phase-based Operational Deflection Shapes (ODSs) were used to compare the theoretical spatial relative-phase pattern derived from the identified section-specific propagation delays with that measured along the viaduct at 20, 30, and 35 Hz. The experimental and theoretical phase patterns showed close agreement at all analyzed frequencies. The measurements revealed clear differences in the wave-propagation characteristics of the ground beneath the viaduct. The native subsoil exhibited lower apparent propagation velocities and greater vibration absorption than the road-substructure materials. For application to train-induced vibrations, the identified propagation characteristics were reduced to representative, frequency-independent parameters: $v_{NATIV}=146\;m/s$ and $\alpha_{NATIV}=0.05788\;m^{-1}$ for the native-subsoil zone, and $v_{ROAD}=505\;m/s$ and $\alpha_{ROAD}=0.002984\;m^{-1}$ for the road-substructure zone. These parameters were used to construct the spatially variable kinematic excitation. Finite-element simulations were performed for the heterogeneous supporting medium and two homogeneous representations. Structural responses generally differed by approximately 20–30%. The homogeneous native-subsoil assumption generally underestimated the heterogeneous-case response, whereas the homogeneous road-substructure assumption overestimated it. These findings demonstrate the importance of accounting for supporting-medium heterogeneity when predicting the response of spatially extended structures to ground-borne vibrations.

## 1. Introduction

Material heterogeneity of subsoil conditions can significantly influence the propagation of vibration waves beneath spatially extended structures such as multi-span viaducts, and consequently lead to non-uniform kinematic excitation. As waves travel through the supporting medium, their velocity, amplitude, and phase change along the propagation path. This problem is especially important for viaducts located at motorway and road interchanges, where different spans may cross different ground zones, including native subsoils, compacted fills, and mechanically stabilized road-substructure materials. These zones may differ in stiffness, density, and wave-propagation properties, which can lead to different propagation velocities and vibration attenuation along the structure. Therefore, realistic modeling of the structural response should account for both spatially variable excitation and material heterogeneity of the supporting medium.

The literature relevant to the present study can be divided into three main thematic areas: spatially varying earthquake ground motion (SVEGM) in bridge structures and multiple-support excitation, the influence of local site conditions and supporting-medium heterogeneity on the response of multiple-support structures, and experimental identification and theory of wave propagation in soils, including the determination of propagation velocities and vibration attenuation.

The first area concerns SVEGM in general, with particular emphasis on its application to bridge structures. Fundamental studies established the roles of spatial correlation, wave passage, and incoherence in spatially variable excitation [1,2,3]. Study [4] shows how dense-array measurements can be used to identify SVEGM parameters directly from recorded ground motions. Bridge-specific studies showed that non-uniform and asynchronous support excitation can significantly modify bridge reactions, modal contributions, displacements, and internal forces compared with synchronous excitation [5,6,7]. Similar effects were reported for cable-stayed, arch, and multi-span bridges, where wave-passage and travelling-wave effects produced noticeable changes in structural response and pier forces [8,9,10]. More comprehensive analyses including local site effects and soil–structure interaction confirmed that SVEGM may either increase or decrease selected response quantities depending on structural configuration and excitation characteristics [11,12,13]. Recent developments in SVEGM modeling have introduced more advanced representations of spatially variable excitation. Gurjar and Basu [14] developed a framework combining conditional simulation of multicomponent spatially varying ground motion, including translational and rotational components, with the seismic performance assessment of bridges. Zhao et al. [15] investigated the nonlinear response of curved bridges under nonstationary spatially varying excitation, considering different propagation velocities, local site conditions, and coherence characteristics. These studies demonstrate the increasing complexity of SVEGM models and the importance of accurately representing spatial variability. A recent review also confirmed that the influence of SVEGM on bridge response is strongly case-dependent [16].

The second area addressed local site effects and experimental evidence. Local site conditions are an important source of spatial variability of ground motion [2,3]. Different soil conditions beneath individual supports can modify both the excitation and the resulting bridge response [15,17,18]. Experimental studies have also identified travel-time delays and apparent propagation velocities from support measurements and demonstrated the effects of spatially variable excitation on bridge response [19,20].

The third area concerned the experimental identification of parameters for non-uniform kinematic excitation models. Cross-correlation and signal-processing methods have been used to determine wave travel times and propagation velocities directly from in situ measurements [21,22]. Surface-wave tests were carried out to identify dispersion, attenuation, site-specific wave velocities, damping, and amplitude–distance relationships [23,24], while multi-point measurements allow time delays between different measurement pairs to be compared to verify the identified propagation pattern [25].

Overall, previous studies show that spatial variability of seismic excitation can significantly affect bridge response. Such excitation may also arise from non-seismic sources, including railway-induced ground vibrations. In heterogeneous ground, differences in wave propagation can produce spatially varying support motions. The practical challenge is therefore to represent these differences in a simple engineering model that preserves the experimentally observed section-specific propagation behavior while allowing its reduction to representative parameters for broadband excitation.

The objective of this study is to determine the influence of subsoil material heterogeneity on the dynamic performance of a multi-span viaduct subjected to spatially variable kinematic excitation. For this purpose, apparent propagation velocities and material absorption coefficients are identified for individual sections of the propagation path using synchronized in situ measurements. Phase-based Operational Deflection Shapes (ODSs) are used to assess the consistency of the identified section-specific propagation delays with the measured spatial phase progression [26,27,28,29]. The experimentally identified propagation parameters are then applied in a train-induced vibration analysis to evaluate the influence of subsoil material heterogeneity on the calculated viaduct response.

The novelty of this study lies in the experimental, section-by-section characterization of wave propagation along a viaduct crossing different supporting-medium zones. Based on this characterization, a section-specific wave-propagation model for a heterogeneous supporting medium is formulated. The study combines cross-correlation-based travel-time identification, amplitude-decay characterization, and relative-phase ODS assessment. A further contribution is the transition from experimental parameters to a reduced frequency-independent engineering representation. This makes it possible to quantify how neglecting supporting-medium heterogeneity affects the calculated response to broadband train-induced excitation.

## 2. Materials and Methods

### 2.1. Non-Uniform Kinematic Excitation of Spatially Extended Structures

#### 2.1.1. Spatial Variability of Kinematic Excitation

Spatially extended structures, such as multi-span bridges, may be subjected to different kinematic excitation at individual supports because vibration waves propagate through the supporting medium with finite velocity. Consequently, the excitation reaches successive supports at different times and may also differ in amplitude and frequency content. Such spatial variability is commonly associated with wave-passage, attenuation, loss of coherence, and local site effects [1,2]. Heterogeneity of the supporting medium may additionally modify the amplitude, phase, and frequency content of the propagating motion [3,17].

For multiple-support structures, these phenomena lead to spatially varying kinematic excitation, in contrast to the conventional uniform-excitation assumption, in which identical support motion is imposed simultaneously at all supports. Differences in support motion affect the dynamic component of the structural response, which results from inertia forces, and introduce an additional quasi-static component resulting from differential support displacements induced by wave propagation [30,31,32]. Such an approach requires the adoption of an appropriate model of spatially variable kinematic excitation.

The simplest deterministic approaches account for the wave-passage effect only [32], whereas more advanced formulations may additionally include loss of coherence and local site effects [3,17,33]. The appropriate level of model complexity depends on the excitation characteristics, the spatial extent of the structure, and the properties of the subsoil.

#### 2.1.2. Model of Non-Uniform Kinematic Excitation Adopted in the Present Study

Among the models of spatially variable excitation, the delayed-replica approach [32] provides a relatively simple engineering representation of vibration propagation along a multiple-support structure. A kinematic motion specified at one location is adopted as the reference signal, and the excitation at successive locations is constructed by introducing the propagation delay and the corresponding change in amplitude. The temporal variation in the excitation along the structure is governed by the wave propagation velocity in the supporting medium, whereas the spatial variation in amplitude results from geometrical spreading and material attenuation.

The delayed-replica model is adopted as the basic propagation framework in the present study. The propagation time $\tau_{ij}$ between two locations i and j is expressed as:

(1) $$\tau_{ij}\;=\;\frac{L_{ij}}{v_{ij}}$$

where:

$L_{ij}$—the propagation distance between locations i and j,

$v_{ij}$—the apparent wave propagation velocity along the corresponding distance.

In addition to the time delay described by Equation (1), the model accounts for the reduction in vibration amplitude with increasing distance from the vibration source, which results from geometrical spreading and material attenuation. For surface-wave propagation, the distance-dependent amplitude is described using the relationship [34]:

(2) $$A\left(r\right)=A\left(r_{0}\right){\left(\frac{r_{0}}{r}\right)}^{n}exp[-\alpha \left(r-r_{0}\right)]$$

where:

A(r)—the vibration amplitude at distance r from the source,

$A(r_{0})$—the vibration amplitude at the reference distance $r_{0}$ from the source,

n—the geometrical-spreading exponent, and for the Rayleigh-wave representation adopted in the present study, n = 0.5,

α—the material absorption coefficient.

Considering both the propagation delay $\tau_{ij}$ and the change in vibration amplitude, the kinematic excitation $u_{j}(t)$ at location j can be represented as:

(3) $$u_{j}(t)\;=\;\eta_{ij}\;u_{i}(t\;-\;\tau_{ij})$$

where:

$u_{i}(t)$—the reference kinematic excitation at location i,

$\eta_{ij}$—the dimensionless amplitude-reduction factor describing the total change in vibration amplitude between the two locations,

t—time [s].

In this formulation, $\eta_{ij}$ includes both geometrical spreading and material damping and can be defined from the adopted distance-dependent amplitude-decay relationship (see Equation (2)).

Application of this simplified model requires information on both amplitude attenuation and wave velocity. A single set of these parameters can provide a reasonable engineering approximation when the medium along the propagation path is approximately homogeneous. However, for spatially extended structures such as multi-span viaducts, the supporting medium may be materially heterogeneous. Different parts of the propagation path may pass through different material zones, such as native subsoils or mechanically stabilized road-substructure materials. Under such conditions, a single propagation velocity and a single material absorption coefficient do not adequately represent the spatial variation in wave-passage and amplitude-decay characteristics.

In the heterogeneous formulation adopted in this study, different inter-support sections are therefore described by section-specific apparent propagation velocities and material absorption coefficients identified from in situ measurements.

### 2.2. Investigated Viaduct, Subsoil Conditions, and Numerical Model of the Structure

#### 2.2.1. Viaduct Structure

The investigated structure is a five-span road viaduct located in Chrzanów, Poland. A detailed description of the geometry and material properties of the viaduct is provided in [35,36]. The structure has an overall length of approximately 163 m. The span arrangement, measured between the abutments and consecutive intermediate supports, is 18.5 m + 23.0 m + 80.0 m + 23.0 m + 18.5 m. The overall structural layout, the principal longitudinal dimensions, and the supporting-medium conditions beneath the individual spans are shown in Figure 1. The supporting-medium conditions differ beneath the individual spans, with the central part of the viaduct crossing the motorway.

The viaduct superstructure consists of two continuous prestressed-concrete longitudinal girders connected by a reinforced-concrete deck slab and transverse members. The central 80 m span differs structurally from the shorter side spans and is additionally supported by two reinforced-concrete arches connected to the deck by a system of steel hangers. The deck is 11.80 m wide, while the spacing between the axes of the two main longitudinal girders is 7.20 m. The superstructure is supported on the abutments and four intermediate reinforced-concrete piers by pot bearings.

The principal dimensions of the deck cross-section are shown in Figure 2.

The arches were constructed from C50/60 concrete, the prestressed longitudinal girders from C40/50 concrete, and the piers and abutments from C30/37 concrete. The steel hangers are made of S460 steel, while Y1860 prestressing steel is used for the tendons in the longitudinal girders.

#### 2.2.2. Subsoil and Road-Substructure Material Conditions

The supporting medium beneath the investigated viaduct is spatially heterogeneous, as illustrated in Figure 1. The sections extending from abutment A1 through supports P1 and P2, and from supports P3 and P4 to abutment A2, pass predominantly through the native subsoil, which consists mainly of low-plasticity clayey soils. In contrast, the P2–P3 section crosses the motorway corridor. In this area, the native soil is overlain by a 2.5-m-thick zone of compacted and mechanically stabilized materials forming the motorway road substructure and engineered fill. These materials differ from the native subsoil in terms of material composition, degree of compaction, and stiffness. This spatial material contrast provides the basis for comparing wave-propagation velocity and vibration attenuation between the native-subsoil and road-substructure sections.

#### 2.2.3. Numerical Model of the Structure

A detailed description of the finite element model of the viaduct (Figure 3), developed in Abaqus FEA [37], is provided in [35,36]. The model reproduces the principal load-carrying components of the actual structure, including the prestressed-concrete longitudinal girders, reinforced-concrete deck slab and transverse members, reinforced-concrete piers and arches, steel hangers, and the bearing system.

Different finite-element formulations were adopted according to the geometry and structural function of the individual components. The longitudinal girders, transverse members, piers, and arches were represented by three-dimensional 8-node brick elements, the deck slab by 8-node continuum shell elements, and the steel hangers by linear truss elements. Pot bearings were represented by connector elements with translational degrees of freedom corresponding to the actual bearing arrangement. The finite element mesh comprised roughly 500,000 elements. The effect of prestressing in the longitudinal girders was represented by an equivalent stiffness based on the relative volume fractions of concrete and prestressing steel.

Soil–structure interaction (SSI) was not explicitly included in the FE model, and the pier bases were assumed to be fixed at the foundation level. The adopted structural model had previously been experimentally validated, showing satisfactory agreement between the calculated and measured natural frequencies and mode shapes (see Section 2.2.4). This provides a basis for its application in the present comparative analysis.

#### 2.2.4. Experimental Validation and Dynamic Properties of the Numerical Model

The experimental campaign previously conducted on the viaduct, including the identification of its natural frequencies and mode shapes, has been described in detail in [35,36] and is therefore not repeated here. The experimental program included measurements of the structural response to ambient traffic excitation and additional tests aimed at identifying the dynamic characteristics of the viaduct. Operational Modal Analysis (OMA) was used to identify the natural frequencies and mode shapes, which were subsequently compared with the corresponding results obtained from the FE model.

The first four natural frequencies obtained numerically were 1.60, 2.30, 3.48, and 14.31 Hz, whereas the corresponding experimentally identified values were 1.50, 2.48, 3.36, and 14.26 Hz, respectively. These numerical values remain in overall agreement with the experimental results. A detailed experimental–numerical comparison, including the identified natural frequencies, mode shapes, and their validation, is presented in [36].

The damping properties of the viaduct were also determined experimentally in the previous investigation. Free-decay responses obtained from an in situ excitation test gave logarithmic decrements of 0.13 and 0.17 for the first two vertical vibration modes, corresponding to damping ratios of 0.021 and 0.027, respectively.

### 2.3. Experimental Setup for Wave-Propagation Identification and ODS Assessment

In the present study, only the measurements relevant to the identification of wave-propagation parameters and to the subsequent phase-based ODS assessment are considered. These measurements involved controlled ground excitation and simultaneous recording of the vibration response at four consecutive bridge supports, P1–P4. The recorded signals were used to identify apparent propagation velocities from measured travel-time delays, to assess vibration-amplitude decay along the propagation path, and to determine experimental Operational Deflection Shapes (ODSs) from the frequency-domain relationships between the measured responses.

#### 2.3.1. Excitation System

Controlled ground vibrations were generated using a vibroseis. The device generates vibrations through the vertical motion of its piston and operates over a frequency range of 8–100 Hz. During the tests considered in the present study, the vibroseis was positioned beneath the first span of the viaduct, between abutment A1 and support P1, approximately 10 m from support P1 (Figure 4).

#### 2.3.2. Measurement System and Sensor Configuration

Vibrations were recorded simultaneously at four consecutive viaduct supports, P1–P4, defining the propagation path investigated in the present study. Three-axis accelerometer sets were installed on the piers immediately above ground level. The arrangement of accelerometer sets at supports P1–P4, as well as the sensor installation on a viaduct pier, are shown in Figure 5. The accelerometer sets recorded the vertical (Z) and two horizontal components of acceleration. In the present study, the vertical acceleration records were used to identify the apparent propagation velocities by cross-correlation and the material absorption coefficients from amplitude decay. The same vertical component was used to construct the experimental phase-based ODS.

Acceleration time histories were recorded simultaneously at all measurement locations. Synchronous recording was essential for the adopted methodology, because both the travel-time delays used to identify the apparent wave propagation velocities and the relative phases used to construct the experimental ODS had to be determined from signals referenced to the same time base. Measurements were performed using high-sensitivity, single-axis piezoelectric ICP^®^ accelerometers (model 393B12; PCB Piezotronics, Depew, NY, USA), with a nominal sensitivity of 10 V/g and a measurement range of ±0.5 g (approximately ±4.9 m/s^2^). The frequency range is 0.1–2000 Hz. The signals were recorded at a sampling frequency of 1024 Hz.

### 2.4. Mathematical Framework

#### 2.4.1. Cross-Correlation-Based Identification of Wave Propagation Velocity

The wave propagation velocities along the viaduct were determined from cross-correlation of the measured acceleration signals. For two measurement points *i* and *j*, the propagation delay was determined using the cross-correlation function [38]:

(4) $$R_{ij}(\tau )\;=\;\int \;x_{i}(t)\;x_{j}(t+\tau )\;dt$$

where $x_{i}(t)$ and $x_{j}(t)$ are the filtered acceleration signals and τ is the time lag.

For narrow-band signals, the cross-correlation function may exhibit multiple ambiguous peaks, which can lead to an incorrect time-delay estimate [39]. Therefore, the search for the propagation delay $\tau_{ij}$ was restricted to the physically admissible time-lag range based on the known propagation direction and plausible travel times [40]. When more than one candidate peak remained, consistency between the available support pairs and excitation sweeps was used as an additional selection criterion. The corresponding apparent propagation velocity $v_{ij}$ along the distance $L_{ij}$ is calculated as:

(5) $$v_{ij}\;=\;\frac{L_{ij}}{\tau_{ij}}$$

Cross-correlation-based travel-time estimation is widely used for in situ determination of wave velocities in soils [21,22]. Because surface-wave propagation in layered soils is dispersive [23,24], $v_{ij}$ is interpreted as an apparent propagation velocity representative of the selected narrow frequency band.

#### 2.4.2. Phase-Based ODS Assessment of Spatial Phase Progression

Operational Deflection Shape (ODS) analysis describes the spatial vibration pattern of a system at a selected frequency under forced-excitation conditions. An ODS is constructed from complex vibration responses measured at multiple locations and contains information on their relative amplitudes and phases. By referencing these responses to a common measurement point, the spatial ODS pattern can be expressed in terms of relative amplitude and phase [26,27]. Unlike a mode shape, an ODS generally represents a combination of structural modes; only near resonance may it be dominated by the corresponding mode shape [41]. In the present study, experimental and theoretical phase-based ODSs are compared to assess whether the section-specific propagation delays identified by cross-correlation reproduce the measured spatial phase progression along the structure [27,28].

For a selected frequency f, the complex response $X_{i}\left(f\right)$ at measurement location i is written as [42,43]:

(6) $$X_{i}(f)\;=\;A_{i}(f)\;e^{j\varphi_{i}(f)}$$

where:

$A_{i}(f)$ is the response amplitude;

$\varphi_{i}(f)$ is the response phase angle.

The complex Operational Deflection Shape $ODS_{C}\left(f\right)$ is formed by the set of complex responses $X_{i}(f)$ at all m measurement locations and can be represented by the response vector:

(7) $$ODS_{c}\left(f\right)=\left[\begin{array}{c} X_{1}\left(f\right)\\ X_{2}\left(f\right)\\ \vdots \\ X_{m}\left(f\right) \end{array}\right]$$

For phase-based ODS analysis, one measurement location is selected as a common phase reference. For general notation, the reference location is assigned index 1. The phase ${\Delta \varphi }_{i}(f)$ at each measurement location i is then expressed relative to the phase $\varphi_{\mathrm{ref}}(f)$ at the reference location:

(8) $${\Delta \varphi }_{i}\left(f\right)\;=\;{wrap}_{\left[-180{}^{\circ},180{}^{\circ}\right]}\left(\varphi_{i}(f)\;-\;\varphi_{\mathrm{ref}}(f)\right)$$

where ${wrap}_{\left[-180{}^{\circ},180{}^{\circ}\right]}$ is the phase wrapping.

The relative phase at the reference location is equal to zero. In the phase-based formulation adopted in the present study, the ${ODS}_{phase}\left(f\right)$ is therefore represented by the vector of relative-phase angles $\Delta \phi_{i}\left(f\right)$ at the considered measurement locations:

(9) $${ODS}_{phase}\left(f\right)=\left[\begin{array}{c} 0\\ \Delta \phi_{2}\left(f\right)\\ \vdots \\ \Delta \phi_{m}\left(f\right) \end{array}\right]$$

In this study, for each sweep–frequency combination, the theoretical phase-based ODS is constructed from the section-specific propagation delays identified by cross-correlation, while the experimental phase-based ODS is obtained from the measured relative phases of the same record. The two ODSs are then compared to assess the consistency between the identified propagation delays and the measured spatial phase progression along the viaduct. For the objective of the present study, agreement between the experimental and theoretical ODSs provides additional phase-based support for the heterogeneous wave-propagation model. The delays associated with the native-subsoil and road-substructure sections should jointly reproduce the measured spatial phase progression along the viaduct.

#### 2.4.3. Computational Implementation and Data-Processing Tools

The experimental acceleration records were processed using MATLAB R2023b (MathWorks, Natick, MA, USA). The data-processing procedures included narrow-band Butterworth filtering, cross-correlation analysis for the identification of propagation time delays and apparent wave propagation velocities, and least-squares fitting of the vibration-amplitude-decay model to determine material absorption coefficients. MATLAB was also used for FFT-based determination of relative phases and the construction and comparison of experimental and theoretical phase-based Operational Deflection Shapes (ODSs). These analyses were performed separately for the selected excitation sweeps and target frequencies of 20, 30, and 35 Hz.

The finite-element modeling and dynamic analyses of the viaduct were performed using Abaqus FEA 2022 (see Section 2.2.3). Python 3.12 was used for post-processing the calculated structural response, including the identification of extreme stress and acceleration values and the calculation of root-mean-square (RMS) values. No machine learning methods were employed in the study.

### 2.5. Train-Induced Reference Excitation

For the practical application presented in Section 4, acceleration records measured 10 m from the railway line during a train passage were adopted as the reference kinematic excitation. The horizontal component was assigned to the longitudinal X-direction of the viaduct and the vertical component to the Z-direction. The corresponding time histories and FFT amplitude spectra are shown in Figure 6. The spectra show significant vibration amplitudes within the 20–50 Hz range, with the main spectral peaks indicated in the figure. This frequency content is consistent with published observations of railway-induced ground vibrations, whose characteristic frequencies depend on train speed, vehicle–track interaction, and local soil conditions [44,45]. The spectral characteristics presented in Figure 6 were later used to define the frequency ranges of the controlled vibroseis sweeps and the target frequencies selected for detailed analysis (Section 3.1).

## 3. Results and Discussion

The experimental identification and subsequent numerical application follow the workflow presented in Figure 7. The input data consist of synchronized acceleration records obtained at supports P1–P4 during controlled vibroseis excitation. After narrow-band filtering at 20, 30, and 35 Hz, cross-correlation analysis is used to identify propagation delays and apparent wave velocities, while amplitude-decay analysis provides the section-specific material absorption coefficients. The identified propagation delays are then assessed using phase-based ODSs by comparing the theoretical and experimental spatial relative-phase patterns. Subsequently, the experimentally identified propagation characteristics are reduced to representative frequency-independent parameters for the native-subsoil and road-substructure zones. These parameters, together with the train-induced reference acceleration records, are used to construct three support-excitation scenarios (NATIV, ROAD, and MIX) for the finite-element analysis presented in Section 4.

### 3.1. Experimental Sweep Records and Selected Target Frequencies

The controlled excitation campaign included several frequency sweeps covering a broad frequency range. The complete dataset was analyzed, while detailed results are presented for 20, 30, and 35 Hz, selected for their relevance to the train-induced vibrations considered in the practical application (see Section 2.5). The sweep ranges partly overlap, providing repeated measurements at each target frequency. Results from individual sweeps were analyzed separately to assess the variability and repeatability of the identified propagation parameters. The sweep–frequency combinations used for the detailed wave-propagation and ODS analyses are summarized in Table 1.

Exemplary acceleration time histories recorded at four successive measurement locations during one of the selected sweep tests are shown in Figure 8. The records illustrate the progressive change in vibration amplitude along the propagation path and provide the experimental signals subsequently used for the identification of propagation velocity, amplitude attenuation, and ODS characteristics.

### 3.2. Experimental Identification of Wave Propagation Velocities

The apparent wave propagation velocity for each subsoil section was identified using the cross-correlation procedure described in Section 2.4.1. The measured acceleration signals were filtered using a narrow-band Butterworth filter [46] with a bandwidth of 0.2 Hz, centered at each target frequency (20, 30, and 35 Hz). This bandwidth was adopted to isolate the vibration components around the selected frequencies from the broader excitation sweeps. The same filtering procedure was applied to all measurement signals and excitation sweeps. Because narrow-band filtering may produce multiple correlation peaks, propagation delays were selected within physically admissible time-lag ranges and checked for consistency between the available measurement pairs and excitation sweeps (see Section 2.4.1). The apparent propagation velocities were then calculated from the identified delays and the corresponding distances between supports.

#### 3.2.1. Cross-Correlation Results for the Individual Sweeps

Two propagation sections were selected to characterize the two supporting-medium conditions (see Figure 1). The propagation velocity $v_{12}$, representative of the native-subsoil zone, was determined from the cross-correlation of the signals recorded at P1 and P2, where the vibration response was strongest. The propagation velocity $v_{23}$, representative of the road-substructure zone beneath the central P2–P3 span, was determined from the cross-correlation of the signals recorded at P2 and P3.

In addition, the cross-correlation between the signals recorded at P1 and P3 was used to determine the effective propagation velocity $v_{13}$ over the combined P1–P3 path. Based on $v_{13}$ and the velocity $v_{12}$ identified for the P1–P2 section, an additional check value $v_{23}(check)$ was determined for the P2–P3 section and compared with $v_{23}.$ This comparison provides an additional consistency check of the propagation times obtained from different measurement pairs, in accordance with multi-point wave-propagation analyses [25]. However, wave reflection, refraction, and mode conversion at the transition between the native subsoil and the road substructure may introduce additional propagation paths and affect the identified time delays.

Figure 9 shows the cross-correlation procedure for the P1–P2 pair at 20 Hz: the filtered acceleration records at P1 and P2 (Figure 9a,b), the corresponding cross-correlation function (Figure 9c), and the selected extremum defining the propagation delay $\tau_{12}$ (Figure 9d). This delay is then used to determine the apparent propagation velocity $v_{12}$.

The same cross-correlation procedure was applied to all remaining sweep–frequency combinations. For each target frequency, the cross-correlation functions with the selected extrema and corresponding time delays are presented.

The results obtained at 20 Hz are shown in Figure 10. The three rows correspond to the P1–P2, P1–P3, and P2–P3 support pairs, used to determine $v_{12}$, the effective velocity $v_{13}$, and $v_{23}$, respectively. The columns represent the individual excitation sweeps. The selected extremum and corresponding propagation delay are indicated in each plot, allowing for direct comparison between the sweeps.

The corresponding results for 30 and 35 Hz are presented in Figure 11 and Figure 12, respectively, using the same arrangement of support pairs and excitation sweeps.

#### 3.2.2. Section-Specific Propagation Velocities in a Heterogeneous Supporting Medium

Table 2 lists the apparent propagation velocities determined by cross-correlation analysis. The velocities $v_{12}$, $v_{13}$, and $v_{23}$ refer to the measurement paths P1–P2, P1–P3 and P2–P3, respectively, and were obtained directly from the signals recorded at the supports. As explained in Section 3.2.1, $v_{13}$ represents an effective velocity over the entire P1–P3 path; therefore, it cannot be regarded as a propagation velocity characteristic of a single material. To verify the results, a control value $v_{23}(check)$ for the P2–P3 section was derived from $v_{13}$ and $v_{12}$ and then compared with the velocity $v_{23}$ obtained directly from the P2–P3 cross-correlation.

At 20 Hz, the identified velocities are highly repeatable across the three sweep series: $v_{12}$ ranges from 134 to 135 m/s, $v_{13}$ from 310 to 311 m/s, and $v_{23}$ from 496 to 500 m/s. At 35 Hz, $v_{12}$ is similarly stable (150–151 m/s), whereas greater scatter is observed for the longer propagation paths.

The largest variability occurs at 30 Hz, particularly for $v_{23}.$ The propagation velocities obtained from the individual sweeps are compared in Figure 13. Within the analyzed 20–35 Hz range, no clear monotonic frequency dependence can be distinguished from the scatter between the individual sweeps. The relatively high apparent propagation velocity of 672 m/s obtained at 30 Hz for the SW4_27–38 Hz sweep may be associated with complex wave-propagation conditions in the layered road-substructure zone. The presence of compacted materials overlying native soil may lead to dispersive and multimodal wave propagation, with different wave components contributing to the measured response. In addition, the narrow-band cross-correlation procedure may introduce ambiguity in the identification of propagation delays. These factors may contribute to the observed sweep-to-sweep variability of the apparent propagation velocity.

A pronounced difference is observed between the two supporting-medium zones. The values of $v_{12}$, associated with the native-subsoil section, are consistently much lower than those of $v_{23}$, associated with the compacted and mechanically stabilized road-substructure materials. This contrast demonstrates that the wave-propagation characteristics differ substantially between the two material zones and supports a heterogeneous description of the supporting medium beneath the viaduct.

The individual sweep–frequency velocities are used in the phase-based ODS assessment presented in Section 3.4.

### 3.3. Experimental Identification of Wave Amplitude Decay

In the adopted amplitude-decay model, the absorption coefficient α represents the material absorption of the supporting medium. The section-specific material absorption coefficients $\alpha_{12}$ and $\alpha_{23}$ were identified for the P1–P2 native-subsoil section and the P2–P3 road-substructure section, respectively, using the amplitude-decay relationship defined in Equation (2).

For each section and each sweep, the response amplitude at the first support was normalized to unity, and the amplitude at the subsequent support was expressed relative to this reference value.

For the P1–P2 section, support P1 was adopted as the reference location at $r_{0}=r_{1}=10\;m$ from the vibration source (see Figure 1 and Figure 4). Since $L_{12}=23\;m$, support P2 was located at $r_{2}=33\;m$. The normalized experimental amplitudes and the corresponding fitted amplitude-decay curve are shown in Figure 14a.

For the P2–P3 section, support P2 was adopted as the reference location at $r_{0}=r_{2}=33\;m$ from the vibration source. Since $L_{23}=80\;m$, support P3 was located at $r_{3}=113\;m$. The corresponding experimental amplitudes and fitted curve are shown in Figure 14b.

For each section, the material absorption coefficient was first determined separately for each excitation sweep, with values ranging from 0.04933 to 0.06821 m^−1^ for the P1–P2 native-subsoil section and from 0.000235 to 0.004719 m^−1^ for the P2–P3 road-substructure section. For individual sweeps, the curve passes exactly through the two normalized amplitude points, giving R^2^ = 1 by construction; this value therefore does not provide an independent assessment of goodness of fit. The final amplitude-decay curves were then obtained by least-squares fitting to the combined experimental data from all sweeps, resulting in representative coefficients of $\alpha_{12}=0.05788\;m^{-1}$ and $\alpha_{23}=0.002984\;m^{-1}$, respectively.

The high R^2^ values (approximately 0.98–0.99) confirm good agreement between the fitted amplitude-decay curves and the measured normalized amplitudes. The absorption coefficient for the native subsoil (α_12_ = 0.05788 m^−1^) was approximately 20 times that for the compacted road-substructure materials (α_23_ = 0.002984 m^−1^), indicating substantially stronger attenuation along the native-subsoil section.

This trend is consistent with published site attenuation data. Athanasopoulos et al. [47] reported attenuation coefficients ranging at 50 Hz from 0.1–0.3 m^−1^ for soft soils to 0.003–0.03 m^−1^ for hard soils, with intermediate values of 0.03–0.1 m^−1^ for competent soils. Amick and Gendreau [48] summarized published absorption coefficients of 0.04 m^−1^ for clayey soils and 0.02 m^−1^ for a concrete slab over compacted granular fill. Their field measurements also showed attenuation coefficients of approximately 0.04–0.07 m^−1^ within the 20–32 Hz frequency range. The present α_12_ value is comparable to these field measurements, whereas α_23_ is approximately seven times lower than the reported value for a concrete slab over compacted granular fill.

The numerical differences may result from variations in material properties, compaction, soil layering, excitation frequency, and measurement conditions. Moreover, the present coefficients were identified from vibrations measured at the viaduct supports rather than directly in the free field. They should therefore be interpreted as effective attenuation parameters representative of the investigated propagation sections rather than intrinsic material constants. Nevertheless, the comparison supports the observed contrast in vibration attenuation between the native-subsoil and road-substructure zones.

### 3.4. Phase-Based ODS Assessment of Section-Specific Wave Propagation

The amplitude-decay component of wave propagation was evaluated separately in Section 3.3, where the measured vibration amplitudes were used to identify the section-specific material absorption coefficients. The purpose of the ODS assessment is different: to evaluate whether the propagation delays identified by cross-correlation are consistent with the measured spatial phase progression along the viaduct. Therefore, only the relative-phase information is considered in the ODS comparison. Including amplitude information would partly duplicate the amplitude-decay assessment and combine the effects of propagation delay and attenuation in a single comparison. The theoretical ODS represents the phase progression resulting from the identified delays, whereas the experimental ODS represents the measured relative phases at supports P1–P4. Agreement between the experimental and theoretical ODS supports the consistency of the section-specific propagation description with the observed spatial phase behavior.

#### 3.4.1. Construction of Experimental and Theoretical Phase-Based ODSs

Following the phase-based ODS formulation presented in Section 2.4.2, the experimental and theoretical phase-based ODSs at supports P1–P4 were compared in the frequency domain. The comparison was performed separately for each target frequency and excitation sweep, with P1 adopted as the common phase reference location.

The experimental phase-based ODS was constructed from the measured phase angles at supports P1–P4, with all phases expressed relative to P1. Thus, the relative phase at P1 is zero, and the experimental phase-based ODS can be written as:

(10) $${ODS}^{EXP}\left(f\right)=\left[\begin{array}{c} 0\\ {\Delta \varphi }_{2}^{EXP}\left(f\right)\\ {\Delta \varphi }_{3}^{EXP}\left(f\right)\\ {\Delta \varphi }_{4}^{EXP}\left(f\right) \end{array}\right]$$

The theoretical phase-based ODS was constructed using the phase shifts predicted by the delayed-replica wave-propagation model defined in Section 2.1.2 and the same phase-reference convention. For the P1–P2 and P2–P3 sections, the propagation delays were calculated using the apparent propagation velocities identified for the corresponding sweep–frequency combination in Section 3.2. Thus, at P2 and P3, the comparison checks whether the propagation delays identified by cross-correlation are consistent with the measured phase progression along the viaduct.

Extension of the theoretical ODS to support P4 required a propagation assumption for the P3–P4 section. Because this section is also characterized predominantly by native-subsoil conditions, its propagation velocity was assumed to be equal to the value identified for P1–P2. This velocity was not identified independently for P3–P4, and possible differences in local subsoil conditions may introduce additional uncertainty into the theoretical phase predicted at P4. Nevertheless, the assumption allows the section-specific phase progression to be extended to P4 while retaining the spatial differentiation between the native-subsoil and road-substructure zones.

The resulting normalized theoretical ODS can therefore be written as

(11) $${ODS}^{THEOR}\left(f\right)=\left[\begin{array}{c} 0\\ {\Delta \varphi }_{2}^{THEOR}\left(f\right)\\ {\Delta \varphi }_{3}^{THEOR}\left(f\right)\\ {\Delta \varphi }_{4}^{THEOR}\left(f\right) \end{array}\right]$$

The phase shifts were accumulated successively along the P1–P4 propagation path so that the theoretical ODS represented the complete spatial relative-phase pattern predicted by the heterogeneous propagation model.

The ODS-based assessment was carried out in two stages. First, the experimental and theoretical ODS relative-phase patterns were compared graphically for all selected excitation sweeps at 20, 30, and 35 Hz. Second, the experimental–theoretical phase agreement was quantified using the local wrapped phase errors and the phase root-mean-square error.

#### 3.4.2. Comparison of Experimental and Theoretical Phase-Based ODSs at Selected Frequencies

The phase-based comparison was performed separately for each target frequency because the theoretical relative phases depend on both the excitation frequency and the section-specific propagation delays. For each sweep–frequency combination, the model-predicted phase pattern was calculated using the corresponding experimentally identified apparent propagation velocities presented in Section 3.2. The experimental and theoretical phase patterns were then evaluated along the complete P1–P4 path, with P1 adopted as the zero-phase reference. The individual sweeps were retained separately to assess the repeatability of the experimental–theoretical phase agreement at each target frequency.

The experimental and theoretical ODS relative-phase patterns obtained at 20, 30, and 35 Hz for all selected excitation sweeps are presented in Figure 15, Figure 16 and Figure 17, respectively.

#### 3.4.3. Quantitative Assessment of Phase Agreement

The ODS patterns shown in Figure 15, Figure 16 and Figure 17 exhibit different degrees of sweep-to-sweep variation depending on the target frequency. The sweep-to-sweep variation is most pronounced at 30 Hz, where the identified propagation velocities show the greatest variability, and least pronounced at 20 Hz, where the velocities are highly repeatable across the individual sweeps (see Section 3.2).

Overall, the comparison in Figure 15, Figure 16 and Figure 17 reveals two main aspects:

(1) Experimental–theoretical agreement: For each sweep–frequency combination, the close agreement between the experimental and theoretical ODS phase patterns indicates that the propagation delays identified by cross-correlation are consistent with the measured spatial phase progression along the P1–P4 path.
(2) Repeatability between excitation sweeps: Comparison of the experimental ODS phase patterns obtained from different sweeps at the same target frequency shows their overall similarity, indicating good repeatability of the measured spatial phase response, although greater variability is observed at 30 Hz.

To quantify the experimental–theoretical phase agreement, the graphical comparisons presented in Section 3.4.2 were supplemented by two complementary measures: (1) the local wrapped phase error, which quantifies the discrepancy at each non-reference support; and (2) the phase root-mean-square error, which expresses the overall magnitude of the phase mismatch. In the following equations, i=2,3,4 denotes the non-reference supports P2, P3, and P4, respectively.

The local phase error $e_{\varphi ,i}(f)$ at the *i*-th non-reference support and frequency f was defined as the absolute value of the wrapped experimental–theoretical phase difference:

(12) $$e_{\varphi ,i}(f)\;=\;\left|{wrap}_{\left[-180{}^{\circ},180{}^{\circ}\right]}\;[{\Delta \varphi }_{i}^{EXP}(f)\;-\;{\Delta \varphi }_{i}^{THEOR}(f)]\right|$$

where $\Delta \varphi_{i}^{THEOR}\left(f\right)$ and $\Delta \varphi_{i}^{EXP}\left(f\right)$ are the theoretical and experimental relative phases, respectively, at support i with respect to the reference support P1 at frequency f.

The three local phase errors at the non-reference supports P2–P4 were combined into a single global measure. The phase root-mean-square error $RMSE_{\varphi }\left(f\right)$ at frequency f was defined as:

(13) $$RMSE_{\varphi }\left(f\right)=\sqrt{\frac{1}{3}\sum_{i=2}^{4}{\left[e_{\varphi ,i}\left(f\right)\right]}^{2}}.$$

To express the global phase discrepancy relative to the maximum possible absolute wrapped phase difference, the normalized percentage root-mean-square error $RMSE_{\varphi ,\%}\left(f\right)$ was defined as:

(14) $$RMSE_{\varphi ,\%}\left(f\right)=\frac{RMSE_{\varphi }\left(f\right)}{180^{\circ }}\cdot 100\%.$$

Thus, $RMSE_{\varphi ,\%}\left(f\right)$ represents the global phase mismatch relative to the $180^{\circ }$ reference value, and it should not be interpreted as a percentage error relative to the measured phase itself.

The values of the phase-agreement measures obtained for the analyzed sweep–frequency combinations are summarized in Table 3.

The results in Table 3 confirm the high experimental–theoretical phase agreement observed in Figure 15, Figure 16 and Figure 17. The largest local wrapped phase error is 6.7°, corresponding to 3.72% of the 180° reference value. $RMSE_{\varphi }$ ranges from 1.44° to 5.74°, while $RMSE_{\varphi ,\%}$ ranges from 0.80% to 3.19%. Thus, the global phase discrepancy remains below approximately 3.2% for all analyzed sweep–frequency combinations.

The mean $RMSE_{\varphi }$ values are 3.58°, 3.12°, and 3.74° at 20, 30, and 35 Hz, respectively. No systematic deterioration in the experimental–theoretical phase agreement with increasing target frequency is therefore observed within the analyzed 20–35 Hz range. Although the sweep-to-sweep variability of the identified propagation velocities is greatest at 30 Hz, the phase agreement remains high for all three 30 Hz sweeps.

The local wrapped phase errors listed in Table 3 are summarized in Figure 18.

The normalized global phase mismatch $RMSE_{\varphi ,\%}$ is summarized in Figure 19.

Taken together, the local phase errors and global RMSE values quantitatively support the graphical comparisons presented in Section 3.4.2. The small phase discrepancies show that the section-specific propagation delays identified by cross-correlation are consistent with the relative-phase patterns observed in the corresponding sweep–frequency records. From the perspective of the main objective of this study, the delays associated with the native-subsoil and road-substructure sections provide a coherent heterogeneous description of the measured spatial phase progression. This supports the use of spatially differentiated propagation parameters in the subsequent comparison of heterogeneous and homogeneous excitation scenarios. Such an ODS-based comparison and relative-phase formulation are consistent with previous studies [27,29].

## 4. Engineering Application to Train-Induced Vibrations

The purpose of this section is to demonstrate the effect of supporting-medium heterogeneity on the calculated dynamic response of the viaduct under train-induced excitation. Railway-induced ground vibration is strongly affected by local propagation conditions, including wave velocity and attenuation [24,49], so spatial heterogeneity of the supporting medium may lead to different support motions along the structure.

It is assumed that the train passes beneath the A1–P1 span (see Figure 1) and generates ground vibrations propagating towards successive supports. The controlled vibroseis and the passing train represent different excitation sources. The vibroseis generates localized vibrations, whereas a passing train is a moving, spatially distributed source. Consequently, the generated wave fields may differ in their frequency content, wave components, and attenuation characteristics. Nevertheless, wave propagation is also governed by the properties of the supporting medium. Previous experimental studies have shown that artificial-source measurements can provide useful site-specific propagation information for railway-induced ground vibrations, although source-dependent differences may occur [24].

In the present study, the parameters identified from the vibroseis tests are adopted as effective section-specific propagation parameters for the train-induced vibration analysis. Their application is based on the assumption that the principal propagation characteristics of the supporting-medium zones remain approximately representative for both excitation sources. Moreover, the identification frequencies of 20, 30, and 35 Hz fall within the main frequency range of the adopted train-induced excitation (see Section 2.5). The phase-based ODS assessment confirmed the consistency of the identified propagation delays with the measured spatial phase progression under controlled excitation. These parameters are therefore used to construct the spatially variable train-induced kinematic excitation and to compare the calculated viaduct responses for heterogeneous and homogeneous supporting-medium representations.

### 4.1. Selection of Representative Propagation Parameters for the Engineering Application

The preceding analyses considered the individual sweep–frequency combinations to characterize the variability of apparent propagation velocities and to assess the consistency of the corresponding propagation delays with the measured spatial phase progression. For the broadband train-induced vibration analysis considered here, however, a reduced frequency-independent engineering representation is required. The experimentally identified propagation characteristics are therefore condensed into representative section-specific parameters for the native-subsoil and road-substructure zones.

The representative propagation velocities $v_{NATIV}$ for the native-subsoil sections and $v_{ROAD}$ for the compacted and mechanically stabilized road-substructure zone are treated as effective section-specific parameters that preserve the experimentally observed contrast between the two supporting-medium conditions in the delayed-replica model. They were calculated as the arithmetic means of the corresponding sweep–frequency values listed in Table 2. The resulting mean velocities and ranges for the P1–P2 native-subsoil section and the P2–P3 road-substructure section are summarized in Table 4.

The adopted values are consistent with published field data. Propagation velocities reported for soft cohesive soils are generally of the order of 100–350 m/s [50,51], whereas higher values have been reported for compacted and stabilized engineered fills, including approximately 229–381 m/s [52] and 400–700 m/s [53]. Thus, $v_{NATIV}$ is consistent with values reported for soft cohesive soils, while $v_{ROAD}$ is comparable with those reported for strongly compacted or stabilized fills.

The material absorption coefficients identified in Section 3.3 are adopted directly as representative attenuation parameters. For the native-subsoil and road-substructure zones, they are denoted $\alpha_{NATIV}$ = $\alpha_{12}$ and $\alpha_{ROAD}$ = $\alpha_{23}$, respectively. Their values are summarized in Table 5.

Together, $v_{NATIV}$, $v_{ROAD}$, $\alpha_{NATIV}$, and $\alpha_{ROAD}$ define the two representative frequency-independent parameter sets used in the propagation scenarios presented in Section 4.2.

### 4.2. Propagation Scenarios for the Train-Induced Excitation

Three propagation scenarios were considered:

(1) Homogeneous native-subsoil scenario (NATIV): the representative native-subsoil parameters were applied along the entire propagation path.
(2) Homogeneous road-substructure scenario (ROAD): the representative road-substructure parameters were applied along the entire propagation path.
(3) Heterogeneous scenario (MIX): the representative native-subsoil parameters were assigned to the A1–P1–P2 and P3–P4–A2 sections, whereas the representative road-substructure parameters were assigned to the P2–P3 section, in accordance with the supporting-medium distribution shown in Figure 1.

The supporting-medium configurations adopted in the three propagation scenarios are summarized in Table 6.

The train-induced acceleration records were used as the reference kinematic input (see Section 2.5). For each scenario, the reference record was applied at A1 and P1, in phase in the vertical direction and in antiphase in the longitudinal direction. The motions at P2, P3, P4, and A2 were then generated successively using the delayed-replica model and the propagation parameters assigned to the corresponding sections. At each location, the reference record was time-shifted according to the adopted propagation velocity and scaled in amplitude using the amplitude-decay relationship. Thus, the three scenarios differ only in the spatial assignment of the representative propagation parameters, allowing for a direct comparison of homogeneous and heterogeneous supporting-medium conditions.

### 4.3. Structural Response Quantities and Control Locations

The numerical analyses were performed using the modal dynamics procedure, including all vibration modes with natural frequencies up to 50 Hz. A uniform modal damping ratio of 0.025 was assigned to all included modes. This value was adopted based on experimentally identified damping ratios of 0.021 and 0.027 for the first two vertical vibration modes (see Section 2.2.4). The same damping ratio was retained for the higher modes, for which experimental damping estimates were unavailable.

Time integration was performed with a constant time increment of 0.002 s, corresponding to 500 time steps per second. The kinematic inputs generated for the three propagation scenarios were imposed on the finite-element model of the viaduct described in Section 2.2.3.

Representative exemplary results shown in Figure 20, Figure 21 and Figure 22 include the maximum principal tensile stress above support P3 and at the mid-span of the main span (P2–P3), and the vertical acceleration at the mid-span of the side span (P3–P4). The heterogeneous MIX scenario was adopted as the reference and compared separately with NATIV and ROAD.

The structural response was evaluated at multiple control points distributed along the viaduct, including locations above the supports, at span midpoints, and at intermediate positions within the spans. Figure 19, Figure 20 and Figure 21 present selected representative results: the maximum principal tensile stress above support P3 and at the mid-span of the main span (P2–P3), and the vertical acceleration at the mid-span of the side span (P3–P4).

To document the spatial variation in the response beyond these illustrative examples, RMS and peak values at nine control locations are additionally summarized in Table 7 in Section 4.4.

### 4.4. Comparison and Engineering Interpretation of the Calculated Dynamic Response

Figure 20, Figure 21 and Figure 22 present representative examples of the calculated maximum principal stresses and nodal accelerations, illustrating the differences between the three propagation scenarios. The differences in dynamic response were assessed based on results obtained at numerous control points throughout the viaduct. All three scenarios use the same simplified frequency-independent delayed-replica formulation; therefore, the observed differences reflect the influence of supporting-medium heterogeneity on the calculated response.

Table 7 summarizes RMS and peak values of maximum principal tensile stress at nine control locations, together with percentage deviations in NATIV and ROAD from the MIX reference scenario. Negative values indicate lower responses than MIX, whereas positive values indicate higher responses.

The overall assessment indicated that RMS differences relative to the MIX scenario could be of the order of 20–30% at selected locations, with their magnitude depending on both the control location and the response measure. The MIX scenario generally produced higher responses than NATIV, for which RMS and peak values were approximately 7% lower than MIX on average. The ROAD scenario generally produced the highest responses, exceeding MIX by approximately 13% in RMS and 18% in peak values on average. Locally, these differences reached approximately 32% for RMS and 36% for peak values, with the largest peak deviation occurring above support P4. These results demonstrate a clear spatial variation in the sensitivity of the calculated response to the supporting-medium representation. Thus, the MIX response generally remained between those obtained for NATIV and ROAD.

The results therefore show that supporting-medium heterogeneity affects the spatial variation in support excitation and the calculated viaduct response. Replacing the experimentally identified heterogeneous propagation path with a homogeneous representation may lead to evident differences in the predicted response. The observed response ordering is also consistent with the wave-passage mechanism. Higher propagation velocity reduces inter-support delays and tends to make the support excitation more synchronous [30,31,54]. In the limiting case, the structural response approaches that obtained under uniform excitation [30]. Previous studies have reported that more synchronous or uniform excitation can produce higher dynamic response levels [30,31]. However, this tendency is not universal, because spatially variable excitation may also amplify selected response quantities through pseudo-static effects [54]. A similar sensitivity to the spatial variation in railway-induced excitation was reported by Hua et al. [55], who showed that uniform excitation may misestimate both response levels and the location of the maximum response.

### 4.5. Limitations of the Study

The presented analysis involves several simplifications and limitations, summarized below.

1. Soil–structure interaction (SSI) was not explicitly considered in the numerical analysis. The same fixed-base structural model was used in all three propagation scenarios (NATIV, ROAD, and MIX) to assess the influence of supporting-medium heterogeneity under identical conditions. Accounting for SSI could modify the quasi-static internal forces and stresses by redistributing deformations between the foundations and the superstructure. It could also affect the dynamic response by changing the natural frequencies and effective damping. These effects may increase or decrease the calculated response. Therefore, the results should be interpreted as comparisons obtained under the same fixed-base assumption.
2. Frequency-independent propagation parameters were adopted, although wave propagation in soil may be dispersive. The mean velocities identified at 20, 30, and 35 Hz varied from −8 to +5% for the native subsoil and from −6 to +8% for the road substructure relative to the representative values, corresponding to propagation-time differences of up to 13 and 11 ms, respectively. These variations also reflect identification uncertainties and cannot be attributed to soil dispersion only.
3. Equal propagation velocities were assumed for the P1–P2 and P3–P4 native-subsoil sections. Since the velocity for P3–P4 was not independently identified, this assumption may affect the calculated structural response. However, these effects are expected to be small because both sections have similar general subsoil conditions.
4. Propagation parameters identified under controlled vibroseis excitation were applied to train-induced ground vibrations. The two excitation sources differ in their spatial and temporal characteristics, so the identified parameters may not fully represent wave propagation and amplitude attenuation during train passage. Therefore, the numerical results should be interpreted primarily as a comparative assessment of the influence of supporting-medium heterogeneity rather than as precise predictions of the train-induced structural response.
5. A uniform modal damping ratio was assumed over the analyzed frequency range. Although the adopted damping ratio of 0.025 was based on experimental measurements of the lower vibration modes, the damping characteristics of higher-frequency modes were not identified. Consequently, possible variations in modal damping with frequency may affect the calculated response, particularly in the higher-frequency range. A Rayleigh damping model could account for frequency dependence, but its stiffness-proportional component may also introduce excessive damping of higher modes if not properly calibrated. The same modal damping was therefore retained in all three propagation scenarios to ensure consistent conditions for the comparative analysis.

Considering these limitations, a homogeneous representation of wave-propagation parameters may be an acceptable engineering approximation when the supporting medium is approximately uniform or when differences in propagation characteristics between individual sections have little influence on the structural response. The applicability of this simplification depends on the subsoil conditions, distances between supports, and excitation frequency range. For spatially extended structures crossing zones with substantially different material properties, the proposed section-specific approach is more appropriate, provided that representative propagation parameters can be identified from experimental measurements.

## 5. Conclusions

This study investigated the influence of subsoil heterogeneity on vibration propagation beneath a multi-span viaduct and on the resulting structural response to spatially variable kinematic excitation. In situ measurements were used to identify section-specific propagation velocities and material absorption coefficients, while phase-based ODSs were used to assess the consistency of the corresponding propagation delays with the measured spatial phase progression. The identified characteristics were then reduced to representative, frequency-independent parameters and applied in a train-induced vibration analysis to evaluate the effect of subsoil heterogeneity on the viaduct response. The following conclusions can be drawn from the study:

1. The in situ measurements confirmed the spatial heterogeneity of the propagation path beneath the viaduct. Cross-correlation analysis showed substantially lower apparent propagation velocities for the native-subsoil section than for the compacted road-substructure section. The results obtained at 20, 30, and 35 Hz showed generally good repeatability. The clear contrast between the two supporting-medium zones indicates that a single propagation velocity cannot adequately characterize the entire path beneath the investigated viaduct. However, for structures with approximately homogeneous supporting-medium conditions, a single representative set of propagation parameters may remain an acceptable engineering approximation.
2. Amplitude-decay analysis further confirmed the subsoil heterogeneity. The identified material absorption coefficient was substantially higher for the native subsoil than for the compacted road substructure, indicating stronger vibration attenuation in the native-subsoil zone.
3. The phase-based ODS comparison showed good agreement between the experimental and theoretical phase patterns at 20, 30, and 35 Hz. This confirms that the section-specific propagation delays identified by cross-correlation are consistent with the measured spatial phase progression along the P1–P4 path. The ODS results therefore support the heterogeneous wave-propagation description adopted for the native-subsoil and road-substructure zones.
4. The train-induced vibration example showed that the subsoil heterogeneity identified in the field measurements affected the calculated viaduct response. Relative to the heterogeneous case, the homogeneous native-subsoil assumption tended to underestimate the response, whereas the homogeneous road-substructure assumption tended to overestimate it. These trends are consistent with the identified propagation characteristics: the higher wave velocity and lower material absorption in the road-substructure zone reduce propagation delays and attenuation of support motions, generally leading to higher structural response levels. Differences in dynamic response levels between the scenarios were generally of the order of 20–30%.

Overall, the results show that subsoil heterogeneity is not fully filtered out by the dynamic behavior of the viaduct. Representing the heterogeneous propagation path with a single set of spatially uniform parameters can therefore lead to non-negligible differences in the predicted response. The heterogeneity of the supporting medium should be considered when defining kinematic excitation for spatially extended structures subjected to surface-generated vibrations.

## Figures and Tables

**Figure 1:**
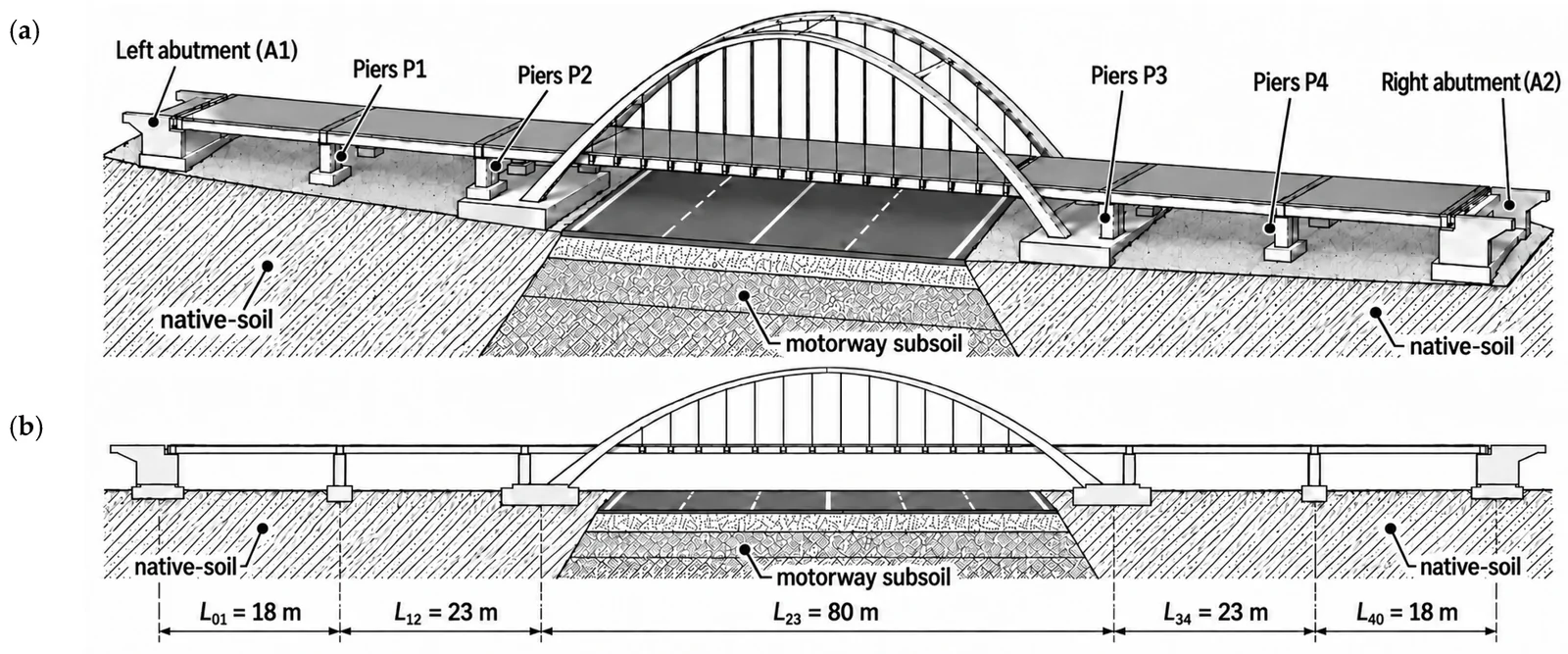
General structural layout of the investigated five-span viaduct: (**a**) general view; (**b**) principal longitudinal dimensions, supporting-medium conditions, and designation of abutments A1 and A2 and intermediate supports P1–P4 used throughout the article.

**Figure 2:**
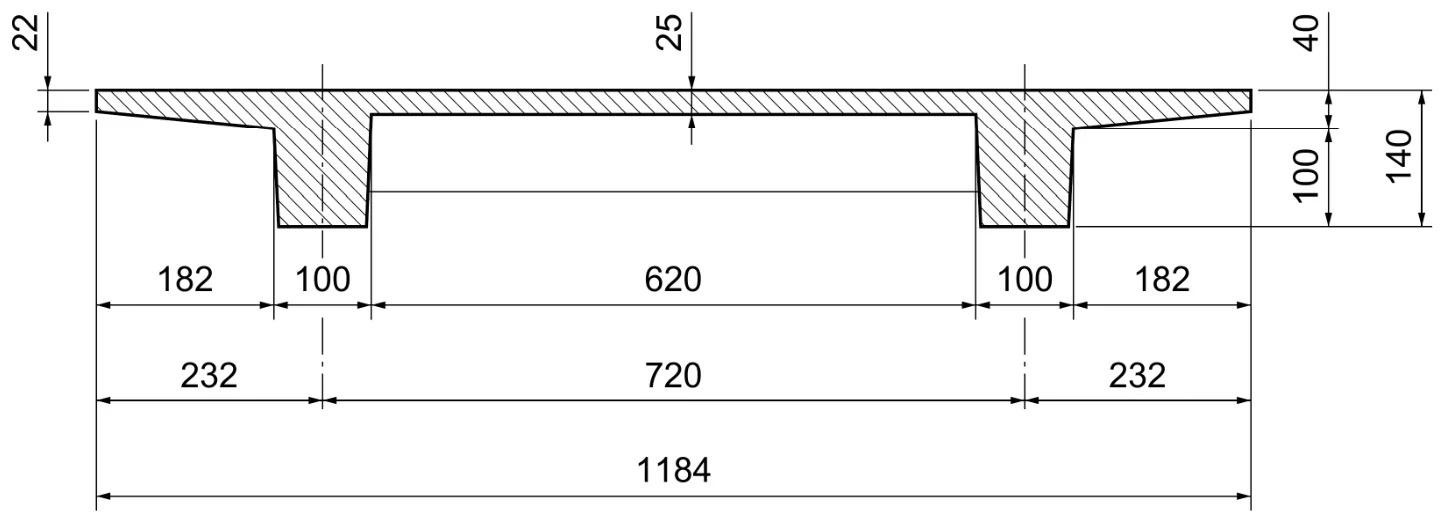
Cross-section of the viaduct deck showing the principal dimensions [cm] and the arrangement of the two longitudinal girders.

**Figure 3:**
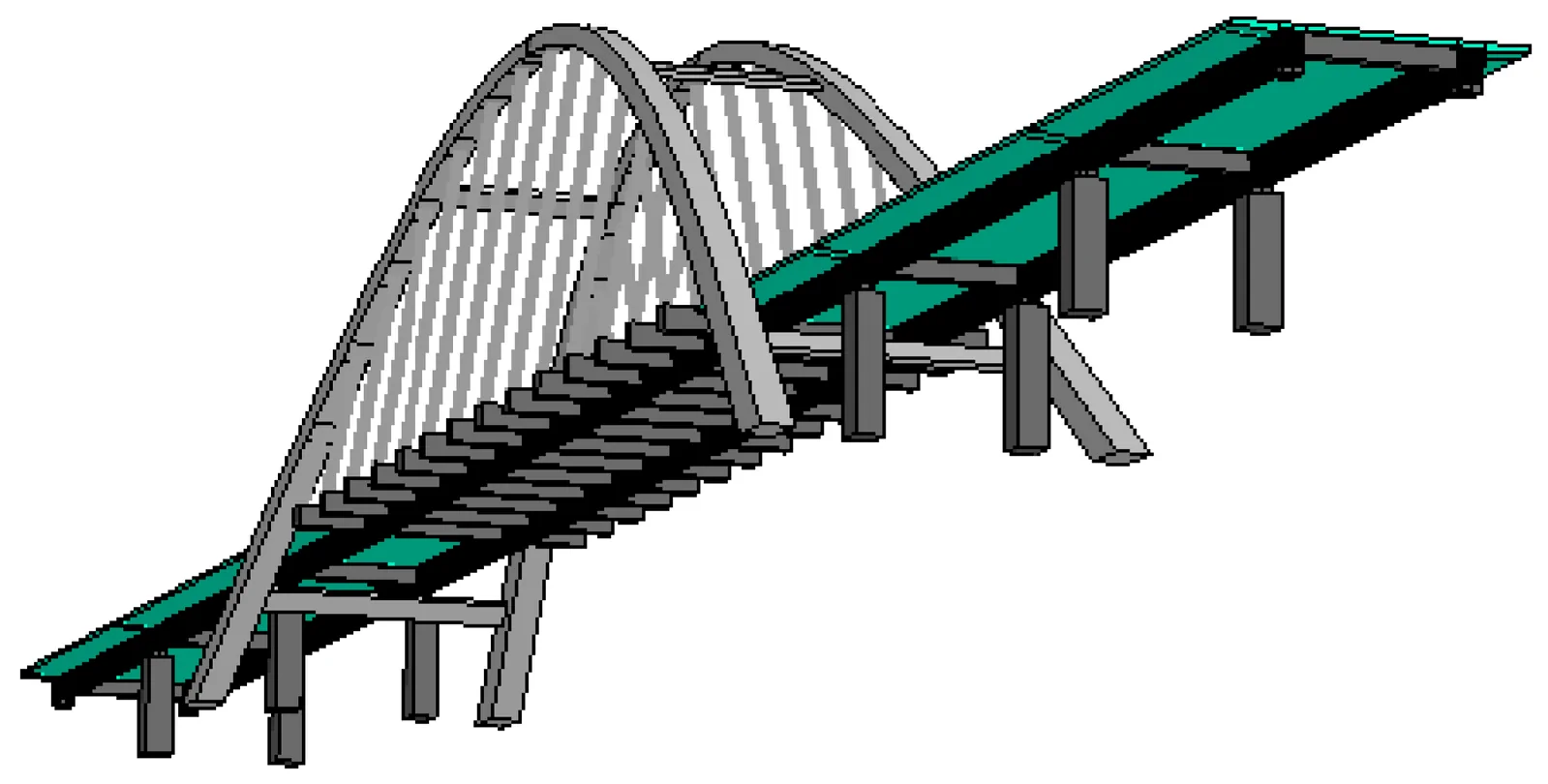
Three-dimensional finite element model of the investigated viaduct.

**Figure 4:**
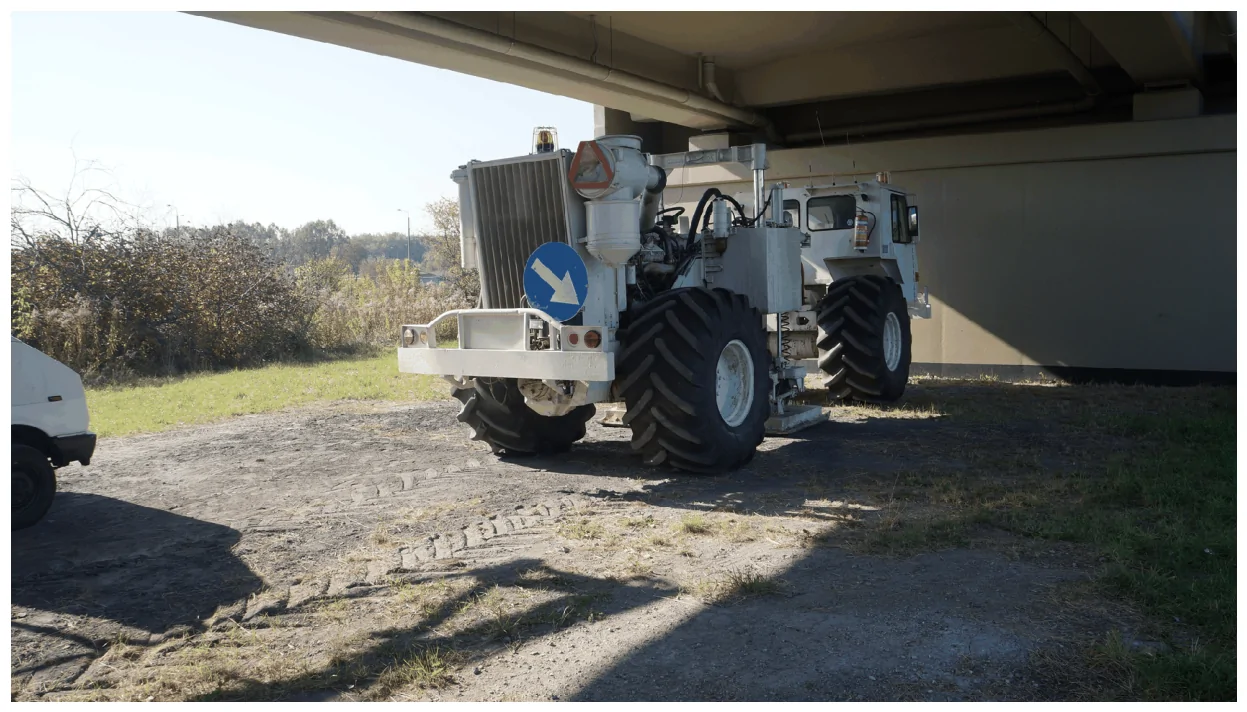
The location of the vibroseis between abutment A1 and support P1 (cf. Figure 1).

**Figure 5:**
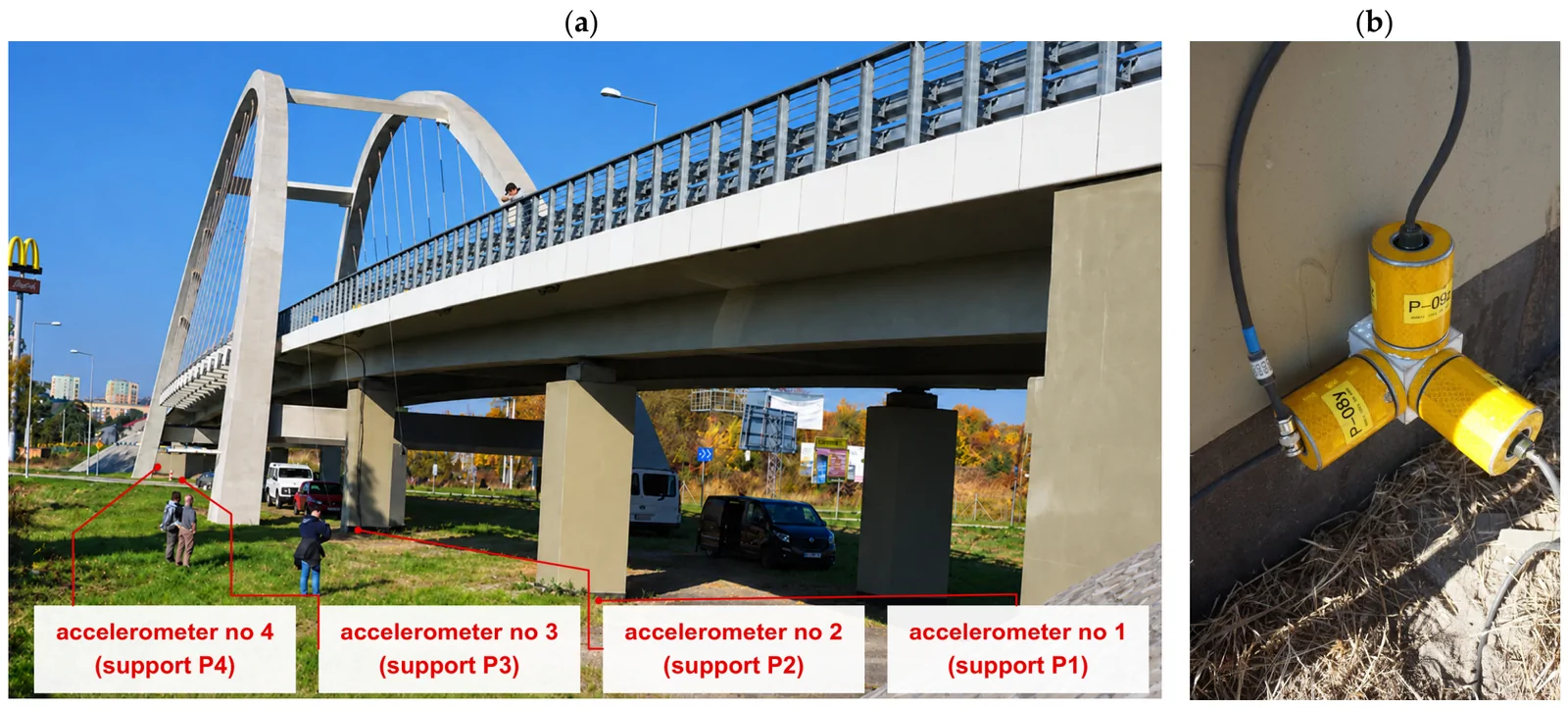
Experimental setup used for the wave-propagation measurements: (**a**) locations of accelerometer sets at supports P1–P4 with the distances of $L_{12}\;=\;23\;m$, $L_{23}\;=\;80\;m,$ and $L_{34}\;=\;23\;m$ between consecutive measurement points; (**b**) accelerometer set placed on the viaduct pier.

**Figure 6:**
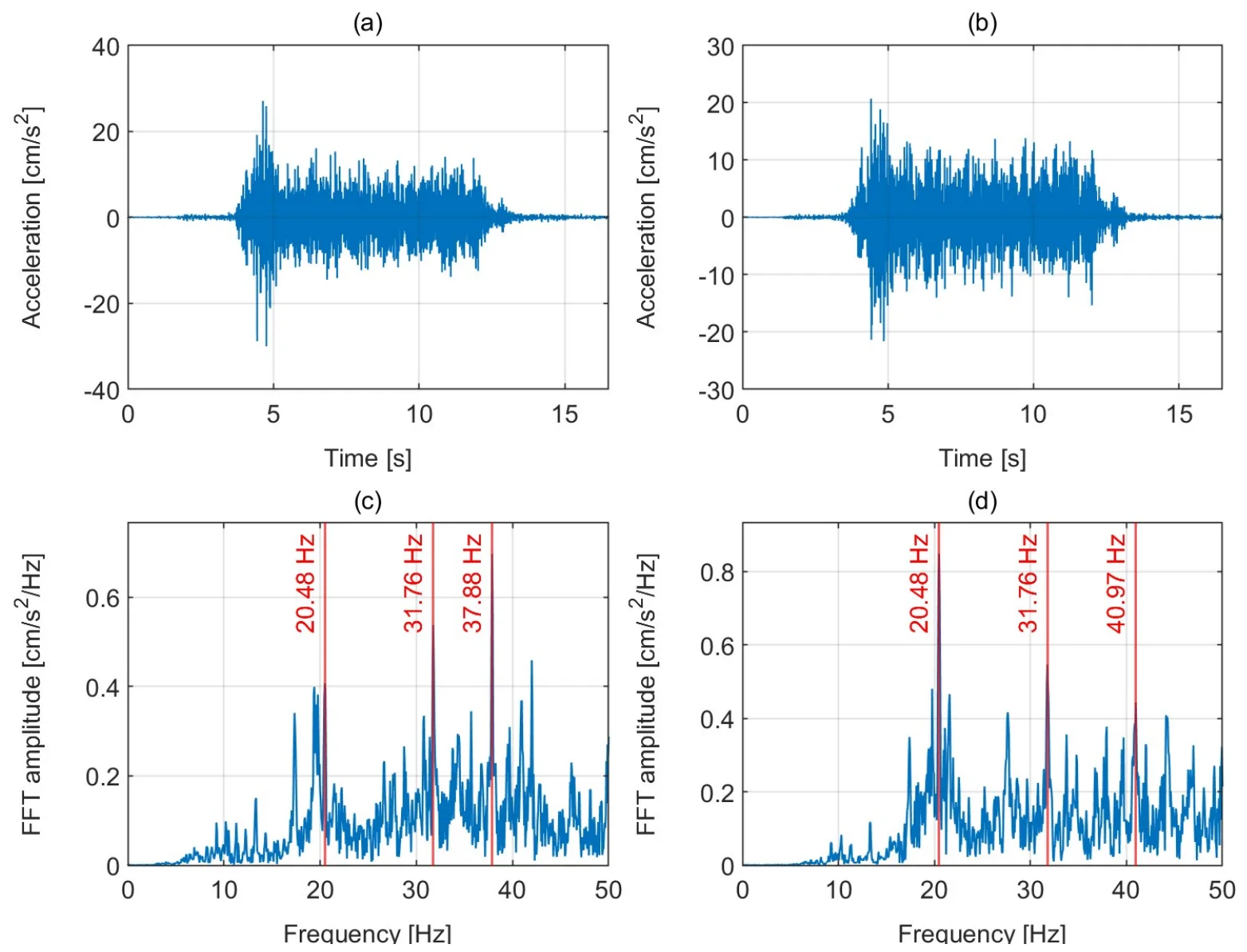
Train-induced acceleration records adopted as the reference kinematic excitation: (**a**) acceleration time history in the longitudinal X-direction; (**b**) acceleration time history in the vertical Z-direction; (**c**) FFT amplitude spectrum of the X-component; (**d**) FFT amplitude spectrum of the Z-component.

**Figure 7:**
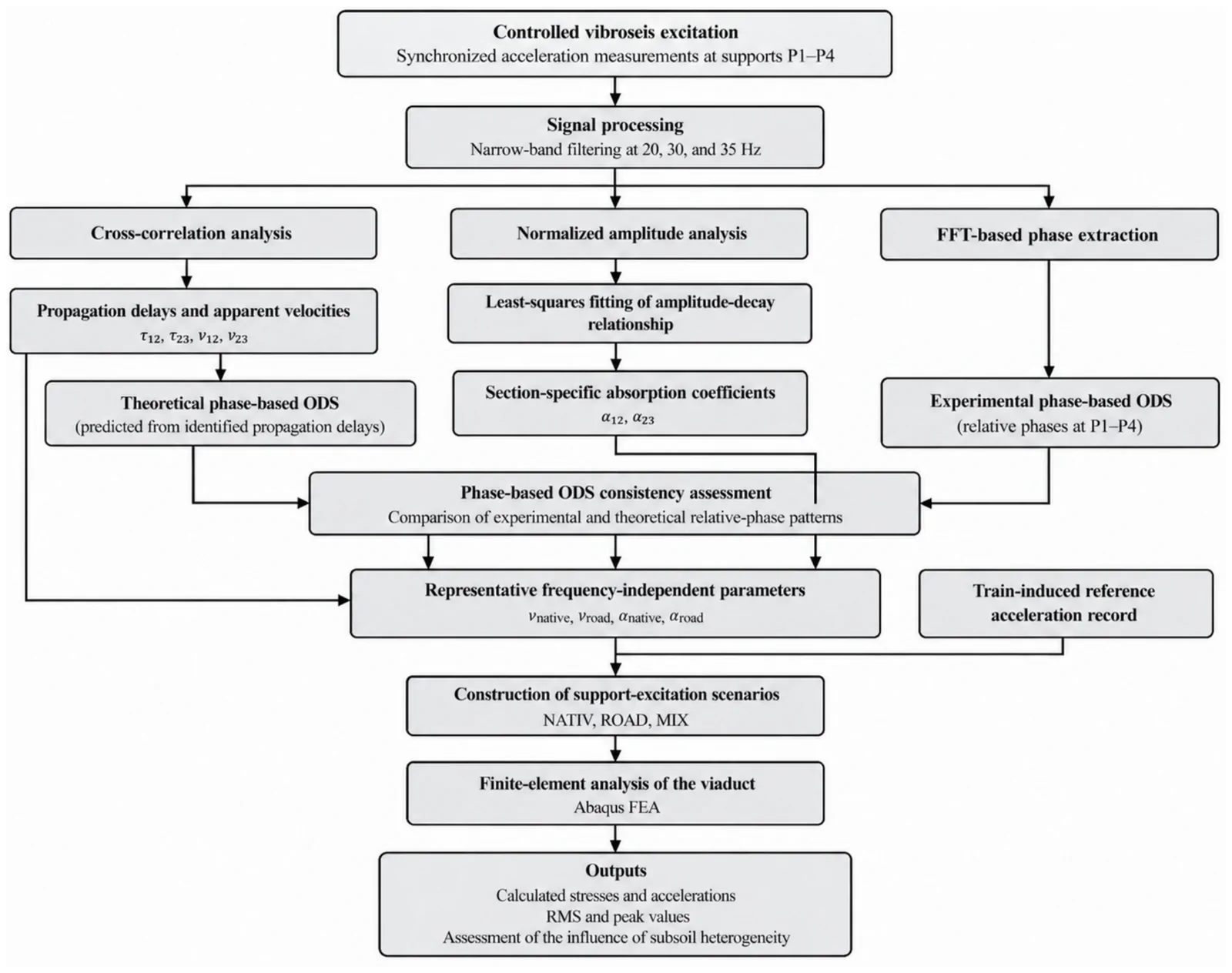
Workflow of experimental identification of wave-propagation parameters, phase-based ODS consistency assessment, and numerical application to train-induced vibrations.

**Figure 8:**
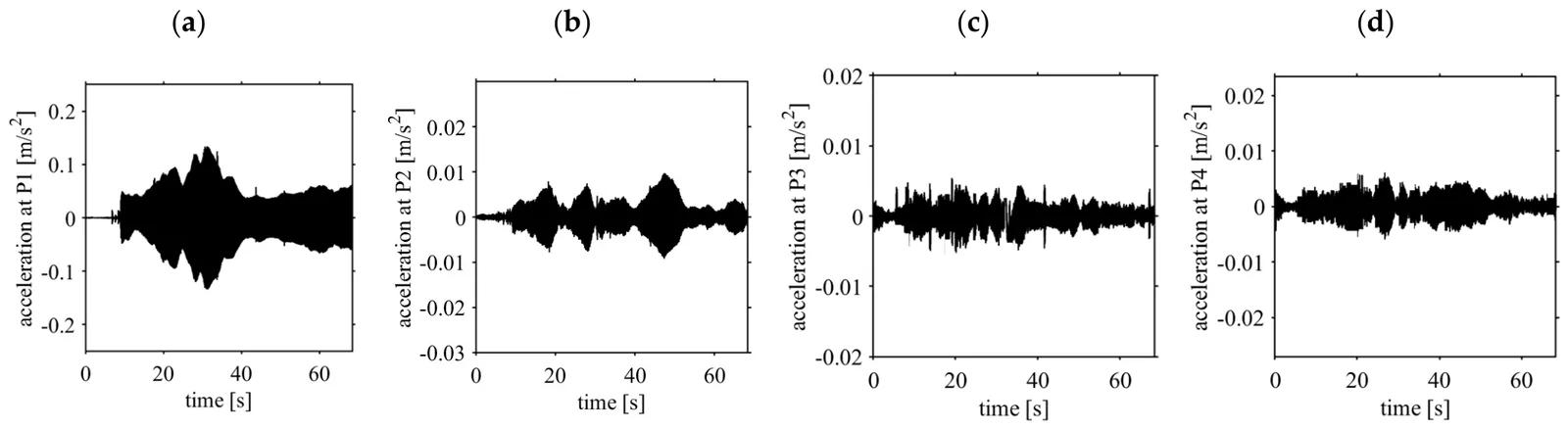
Exemplary acceleration–time histories recorded during a selected frequency sweep at measurement locations: (**a**) P1, (**b**) P2, (**c**) P3, (**d**) P4.

**Figure 9:**
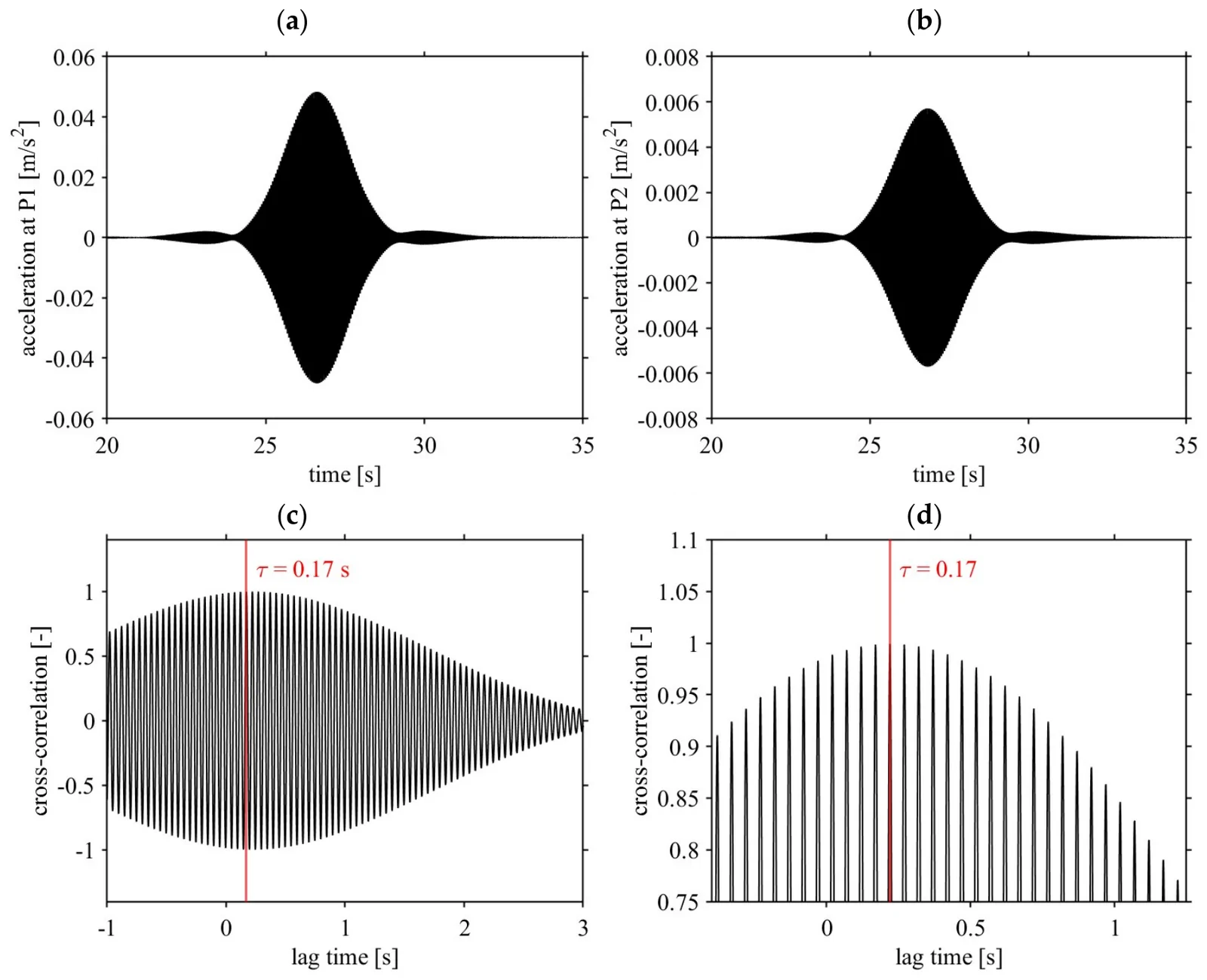
Representative cross-correlation analysis for the 20 Hz component extracted from the SW1_8–50 Hz sweep for supports P1 and P2: (**a**) narrow-band acceleration–time history at support P1, (**b**) narrow-band acceleration–time history at support P2, (**c**) cross-correlation function for the P1–P2 support pair, (**d**) enlarged view of the selected extremum.

**Figure 10:**
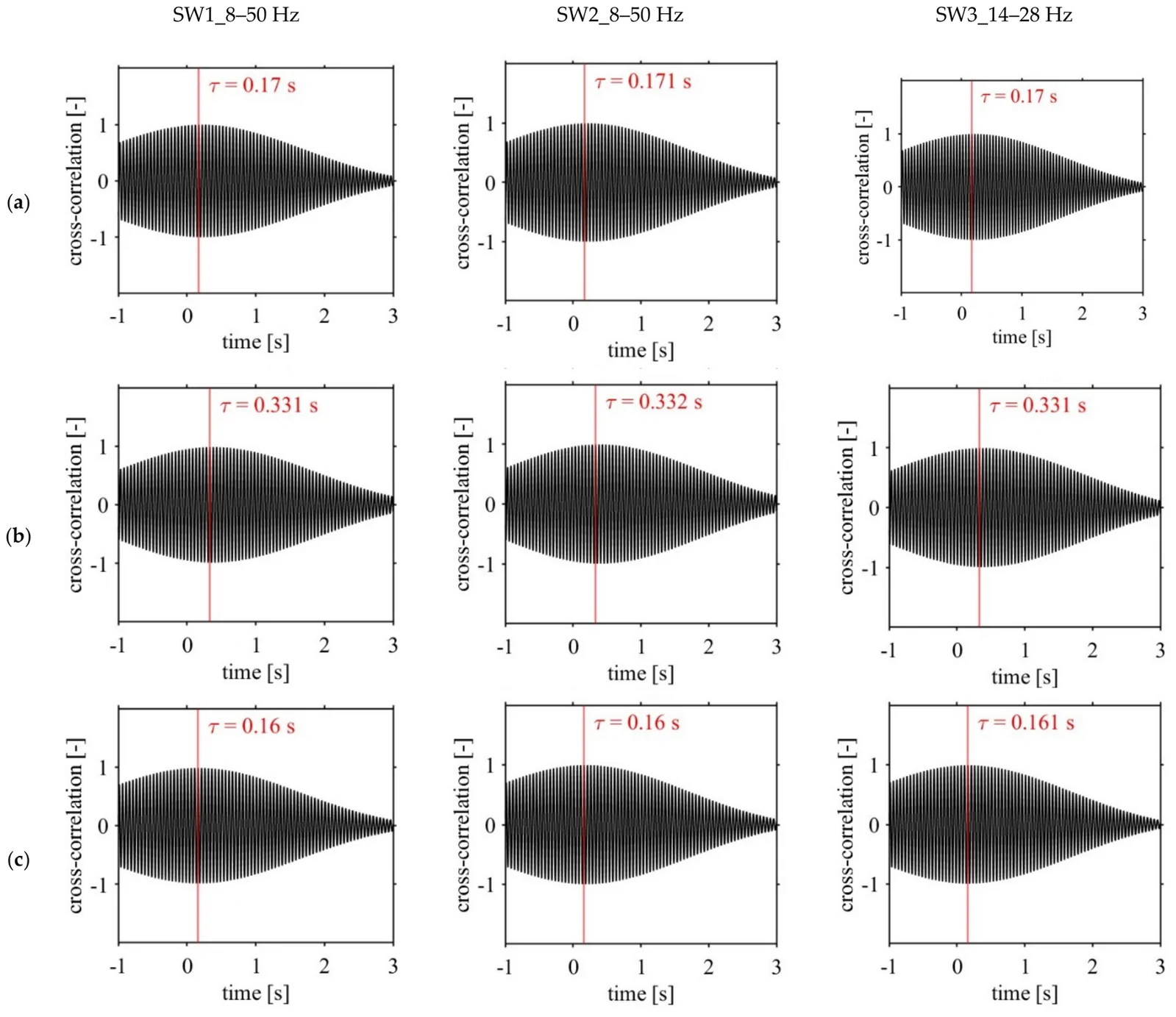
Cross-correlation functions obtained at 20 Hz for the support pairs: (**a**) P1–P2, (**b**) P1–P3, and (**c**) P2–P3 for excitation sweeps SW1_8–50 Hz, SW2_8–50 Hz and SW3_14–28 Hz.

**Figure 11:**
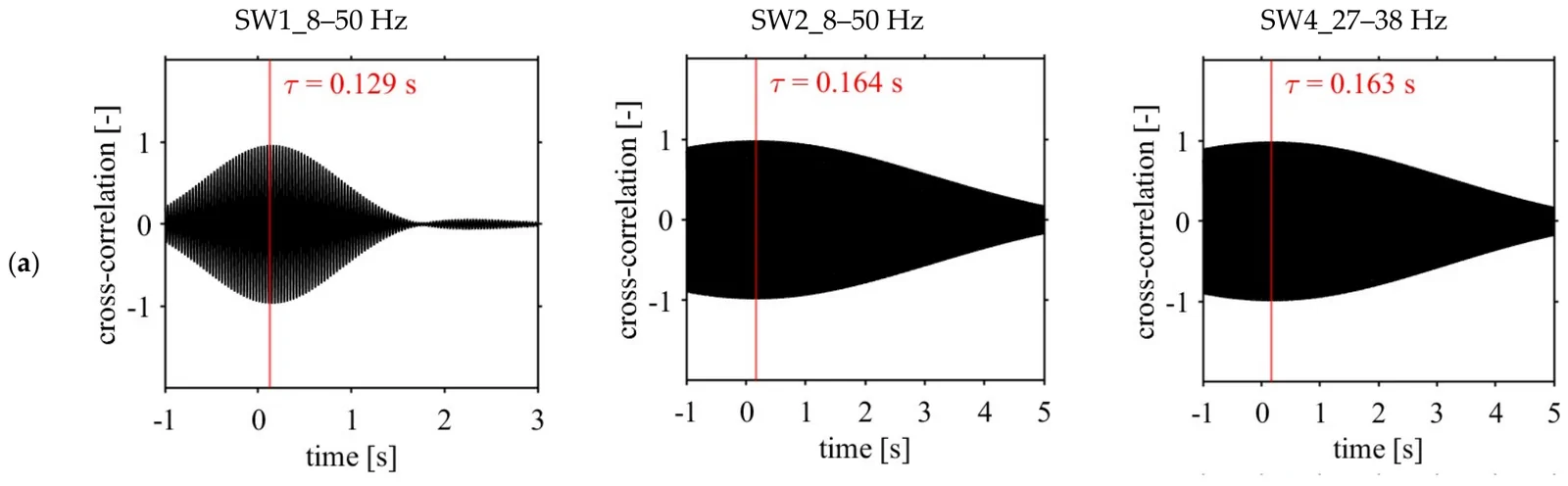

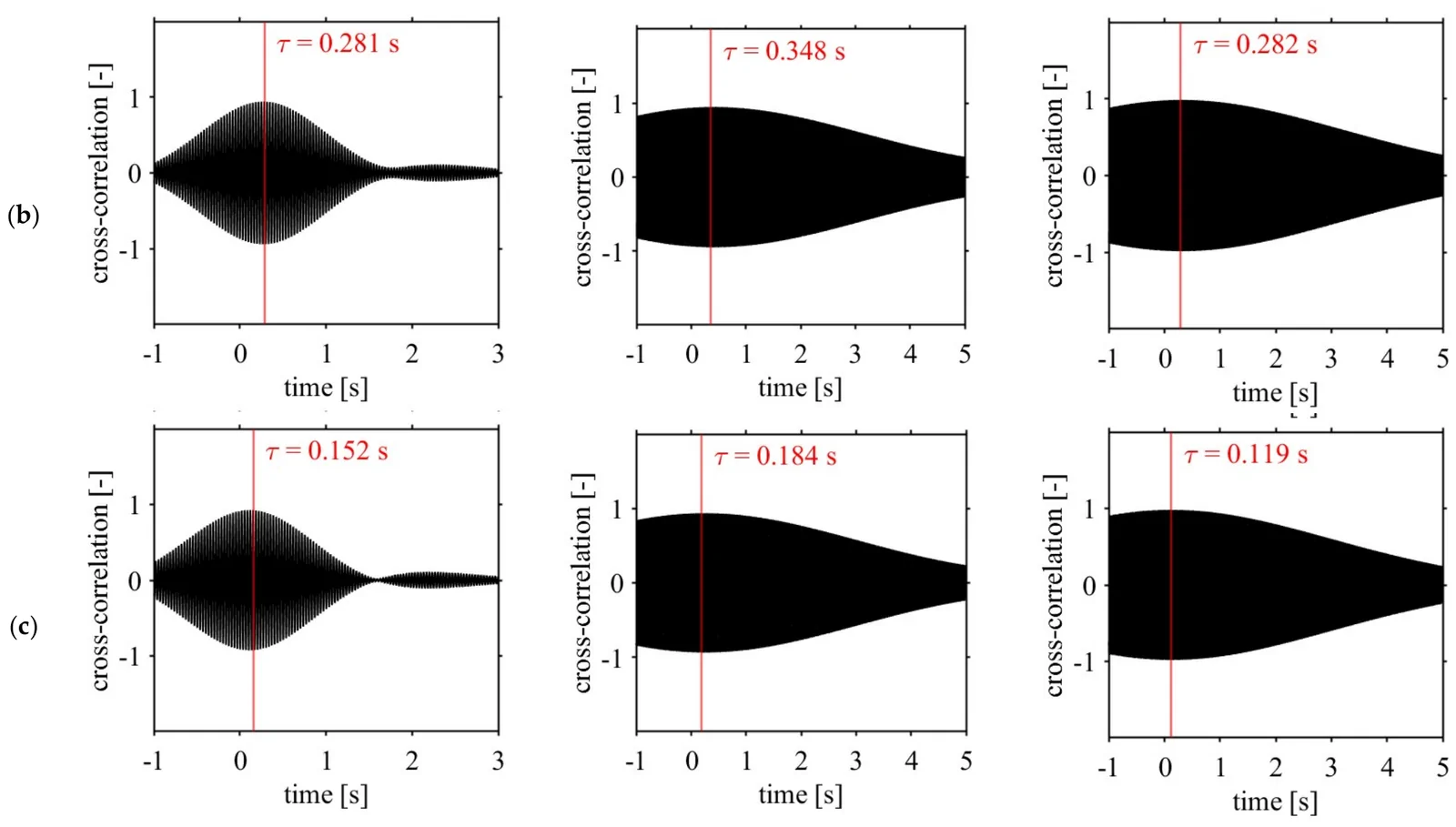
Cross-correlation functions obtained at 30 Hz for the support pairs: (**a**) P1–P2, (**b**) P1–P3, and (**c**) P2–P3 for excitation sweeps SW1_8–50 Hz, SW2_8–50 Hz and SW4_27–38 Hz.

**Figure 12:**
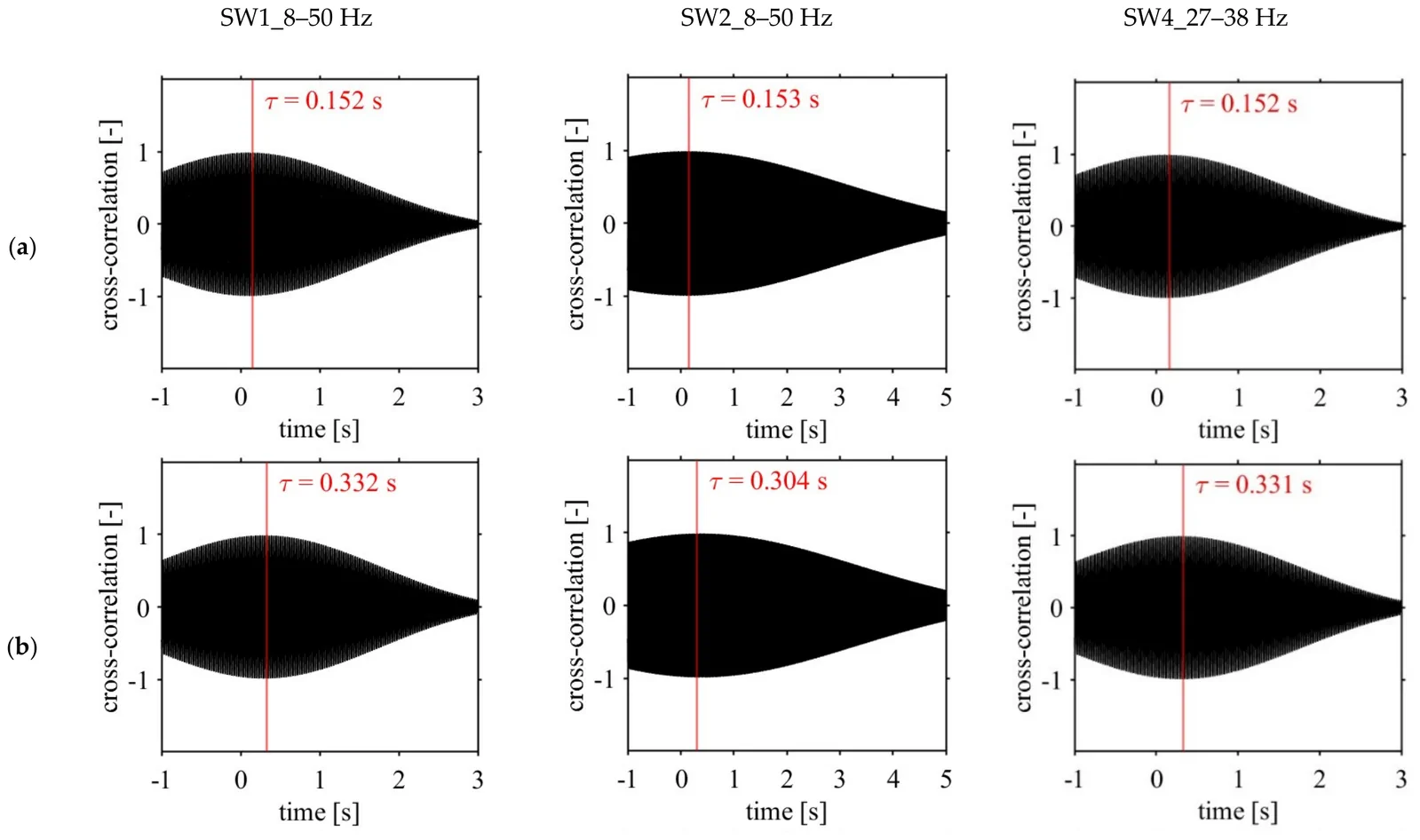

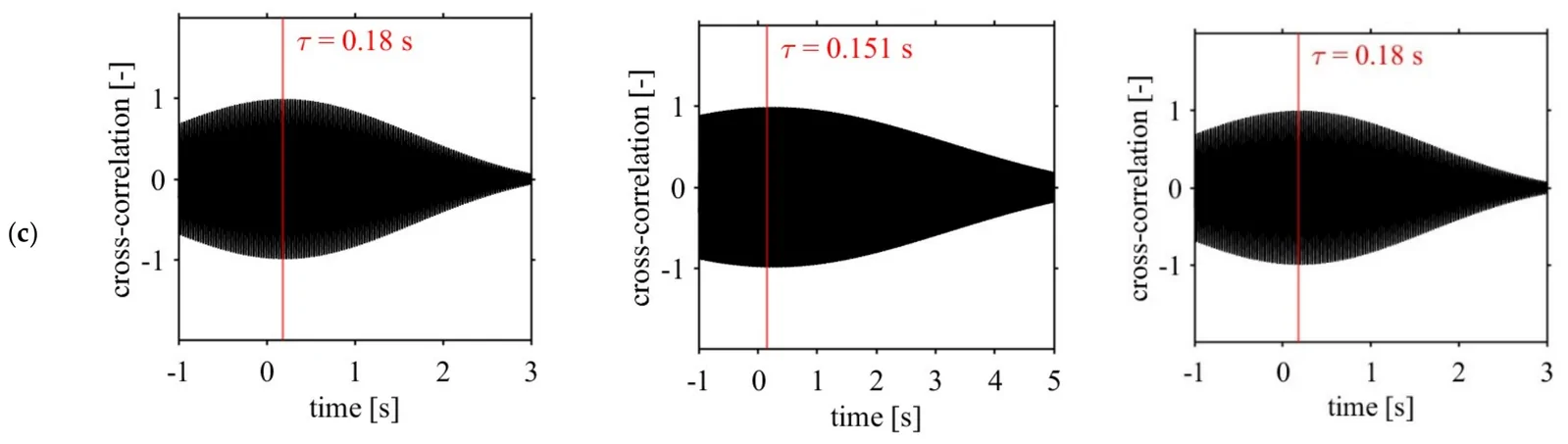
Cross-correlation functions obtained at 35 Hz for the support pairs: (**a**) P1–P2, (**b**) P1–P3, and (**c**) P2–P3 for excitation sweeps SW1_8–50 Hz, SW2_8–50 Hz and SW4_27–38 Hz.

**Figure 13:**
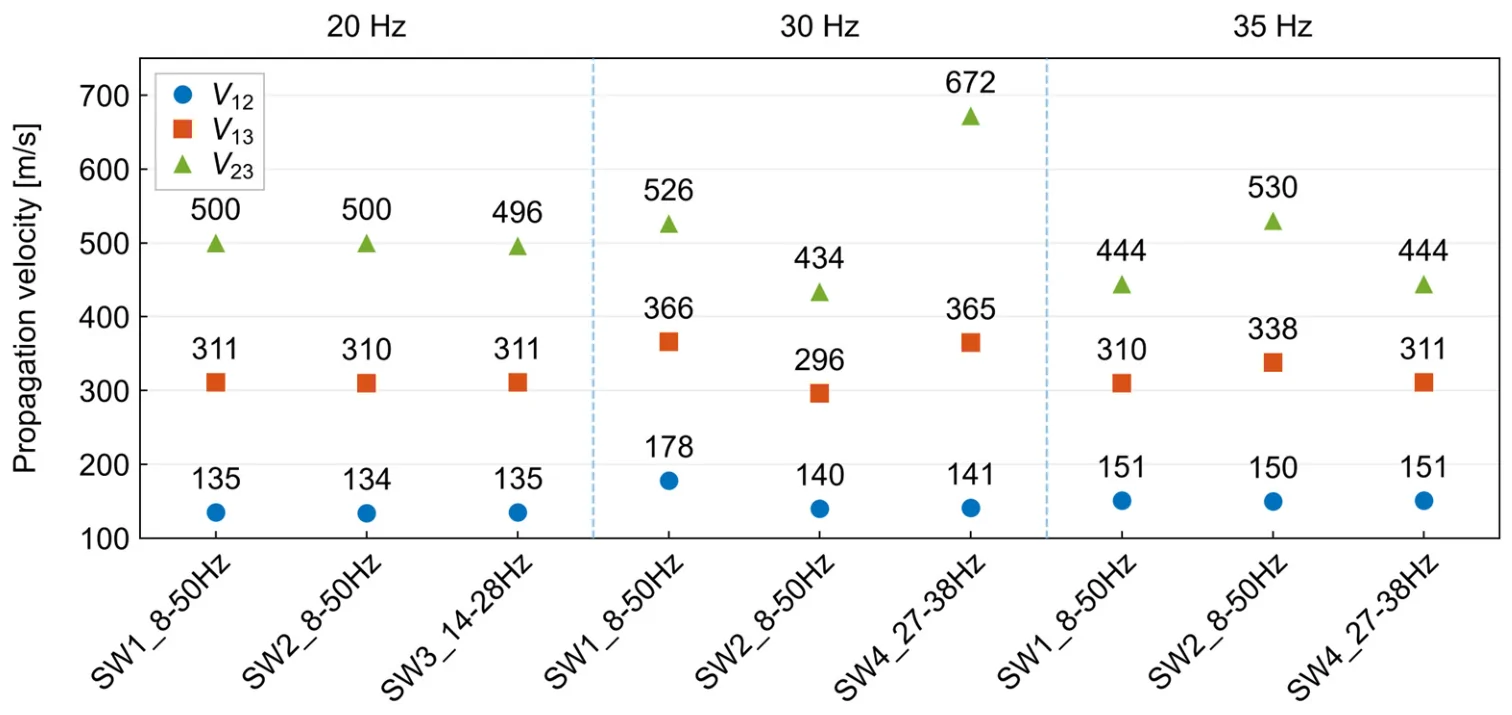
Apparent propagation velocities $v_{12}$, $v_{13}$, and $v_{23}$ identified from the individual sweep series at the target frequencies of 20, 30, and 35 Hz.

**Figure 14:**
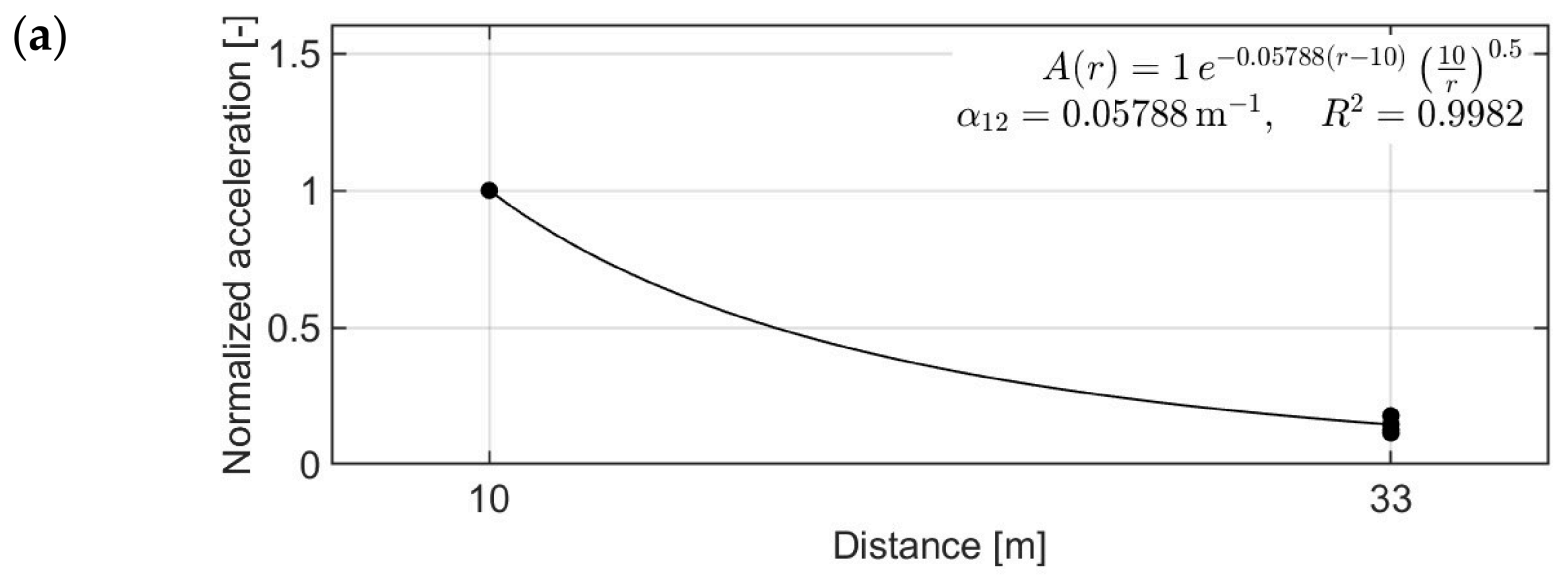

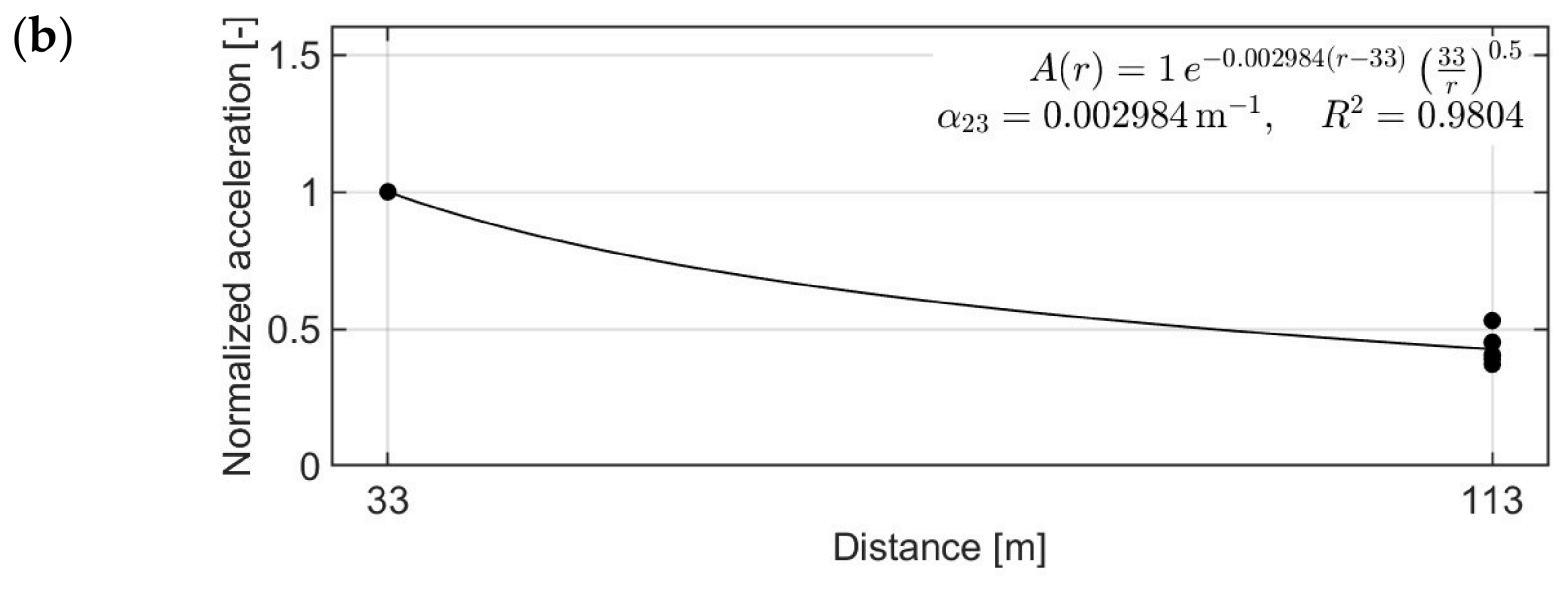
Experimental identification of the section-specific material absorption coefficients from normalized vibration amplitudes for: (**a**) P1–P2 native-subsoil section; (**b**) P2–P3 road-substructure section.

**Figure 15:**
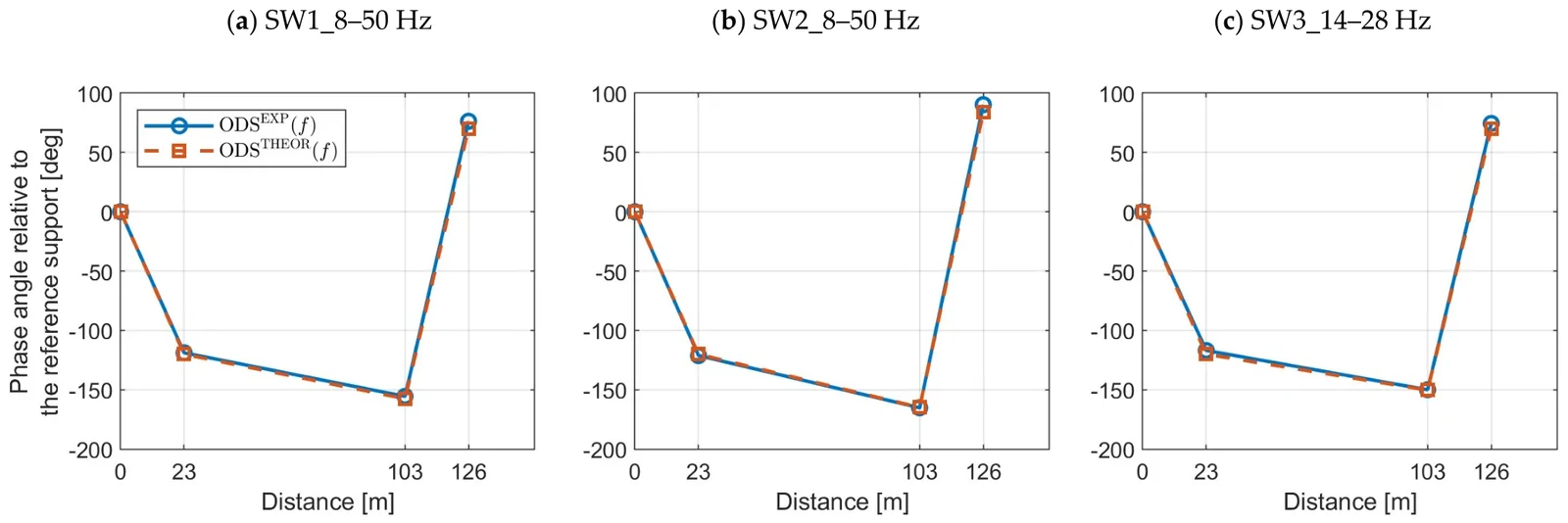
Comparison of experimental and theoretical ODS relative-phase patterns at 20 Hz for the selected excitation sweeps: (**a**) SW1_8–50 Hz, (**b**) SW2_8–50 Hz, and (**c**) SW3_14–28 Hz.

**Figure 16:**
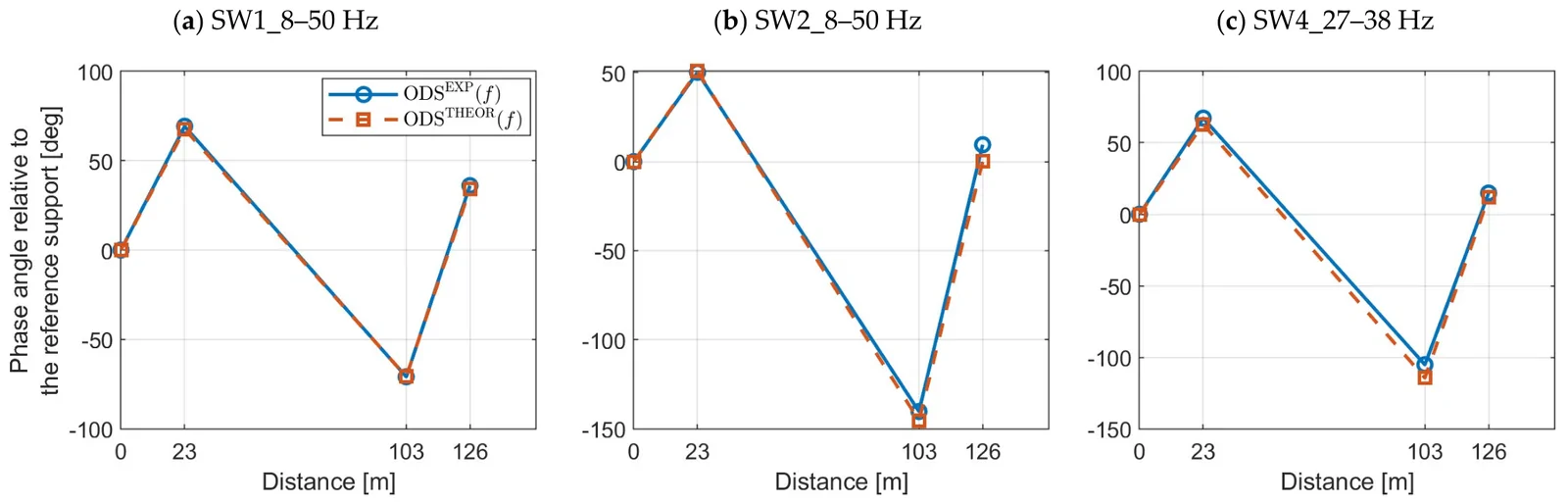
Comparison of experimental and theoretical ODS relative-phase patterns at 30 Hz for the selected excitation sweeps: (**a**) SW1_8–50 Hz, (**b**) SW2_8–50 Hz, and (**c**) SW4_27–38 Hz.

**Figure 17:**
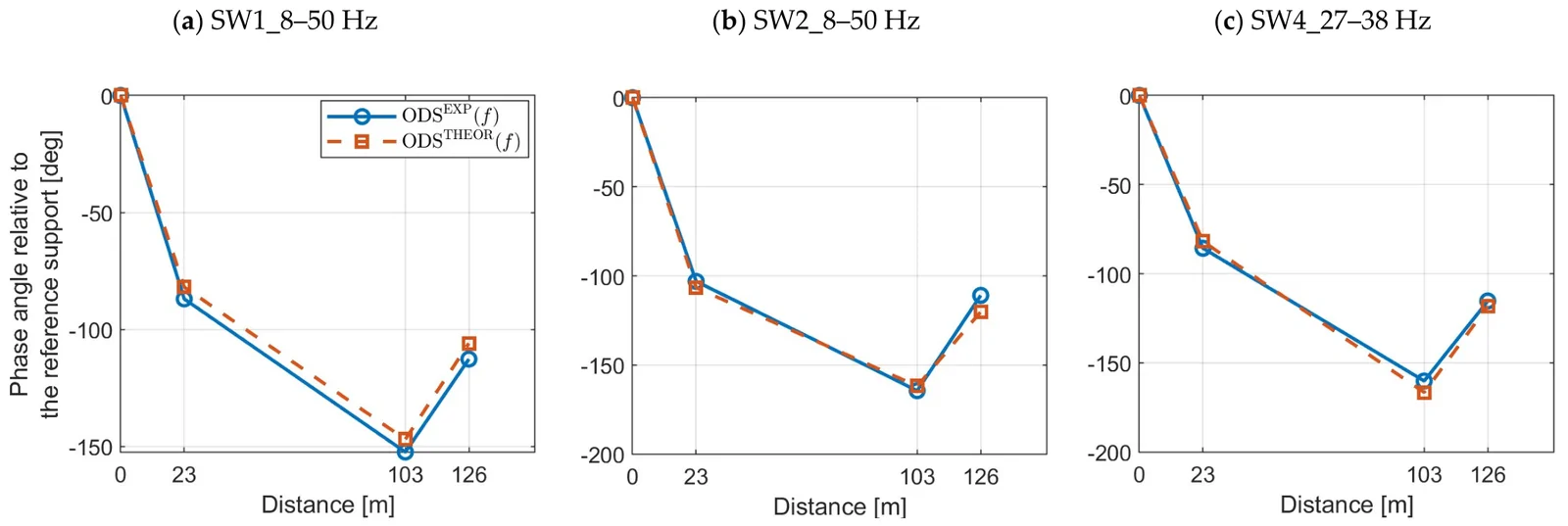
Comparison of experimental and theoretical ODS relative-phase patterns at 35 Hz for the selected excitation sweeps: (**a**) SW1_8–50 Hz, (**b**) SW2_8–50 Hz, and (**c**) SW4_27–38 Hz.

**Figure 18:**
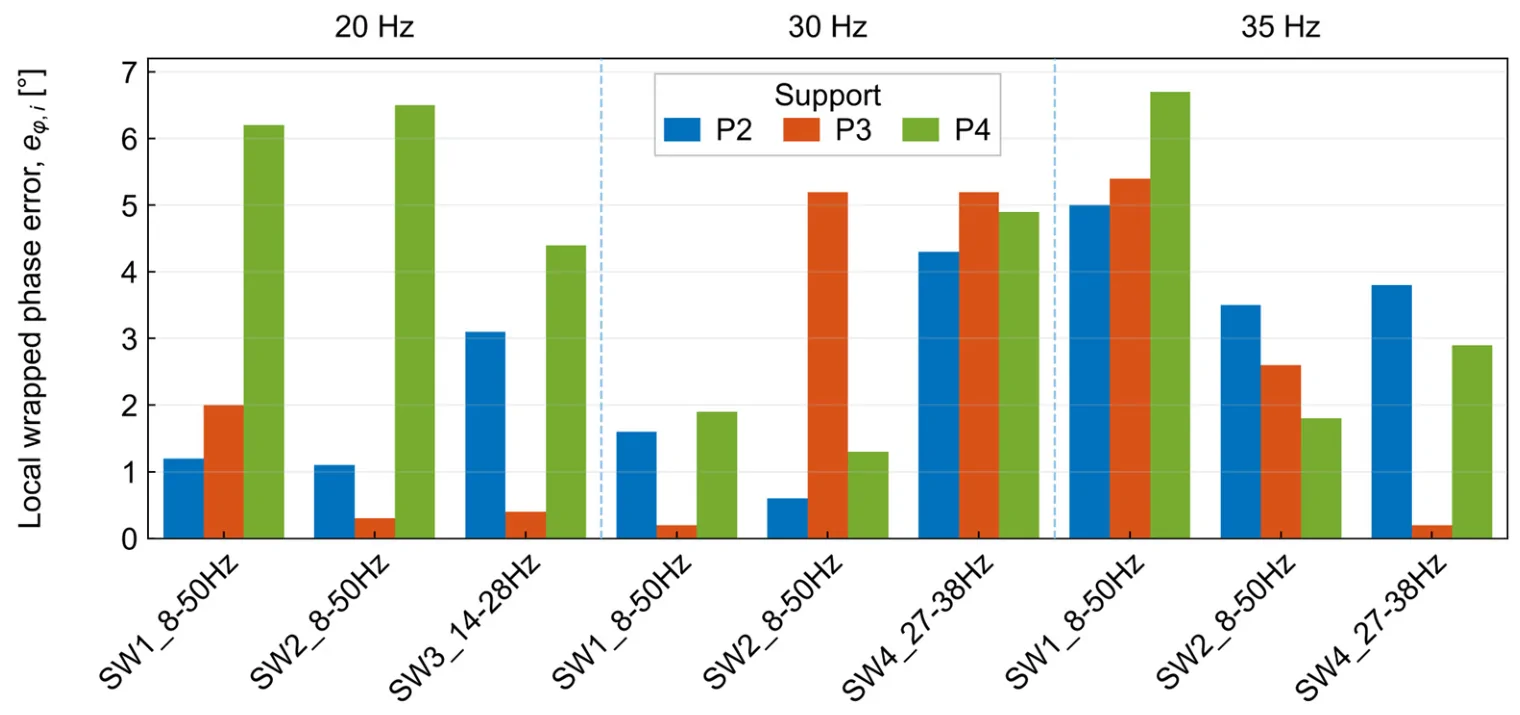
Local wrapped phase errors $e_{\varphi ,i}$ between the experimental and theoretical ODSs at supports P2–P4 for the individual excitation sweeps at 20, 30, and 35 Hz.

**Figure 19:**
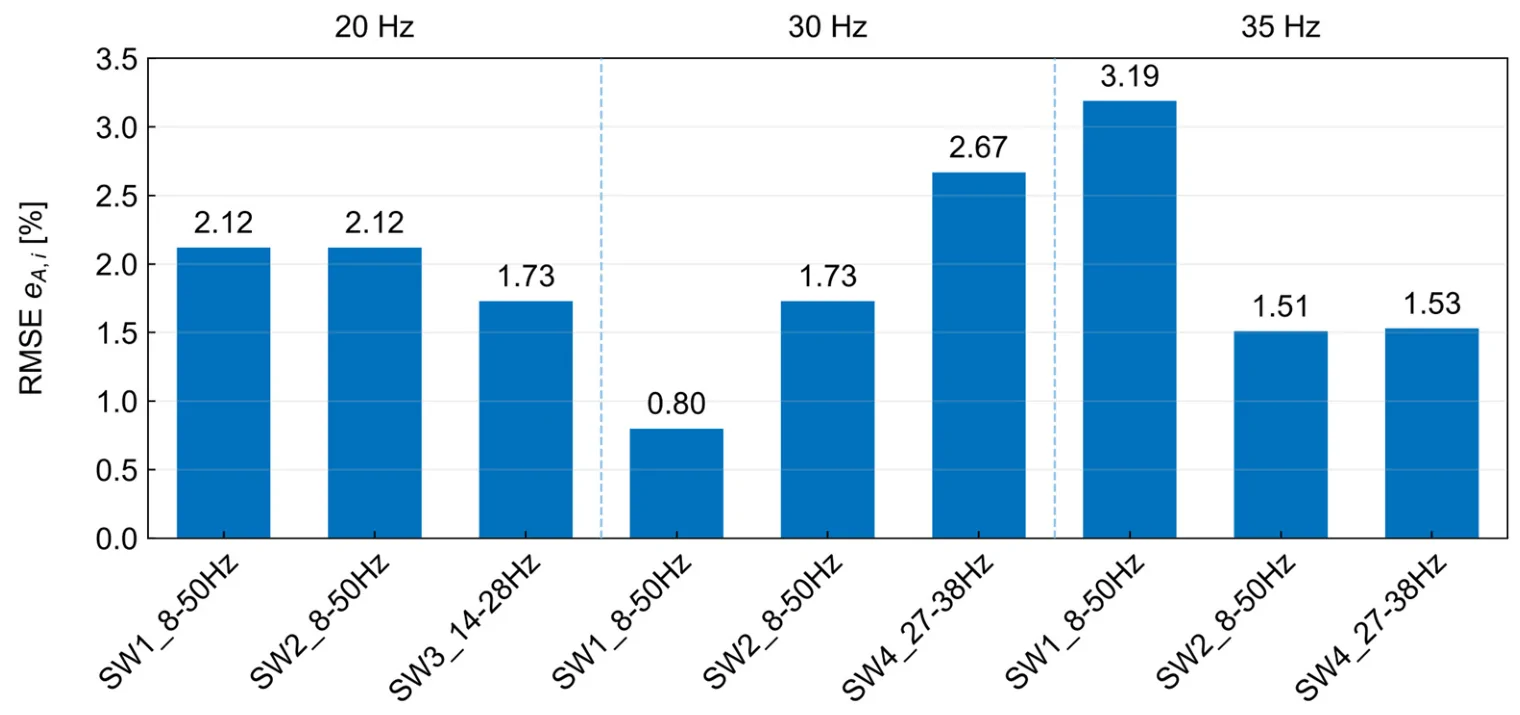
Percentage phase root-mean-square error $RMSE_{\varphi ,\%}$ between the experimental and theoretical ODSs for the selected excitation sweeps at 20, 30, and 35 Hz.

**Figure 20:**
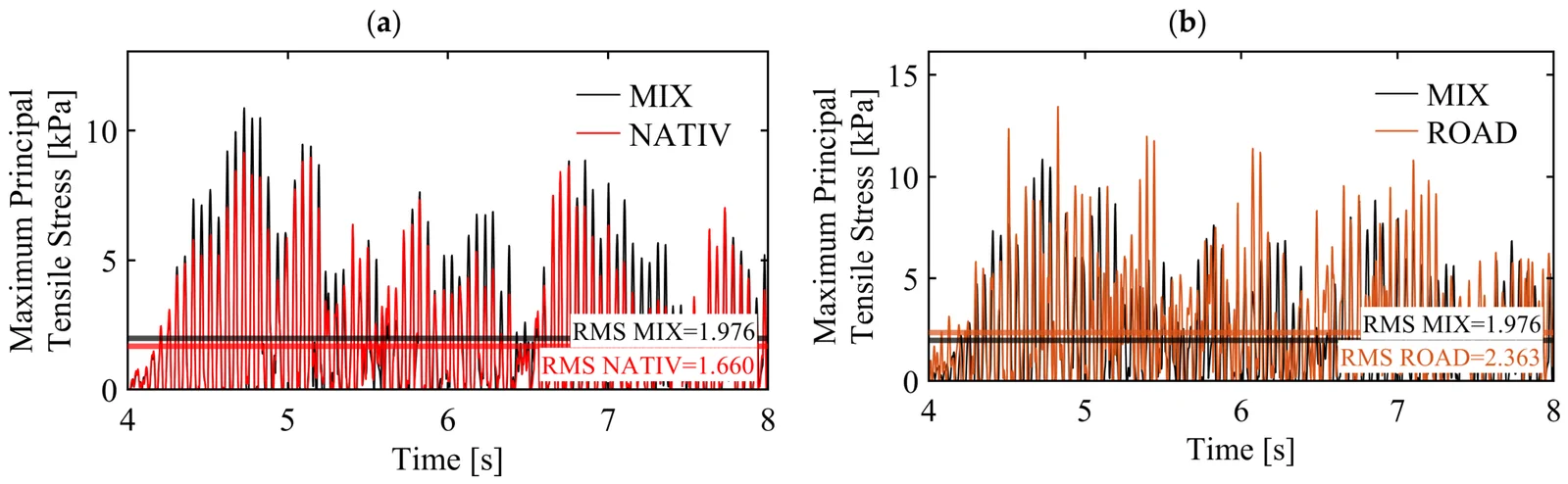
Comparison of maximum principal tensile stress above support P3: (**a**) MIX vs. NATIV, (**b**) MIX vs. ROAD.

**Figure 21:**
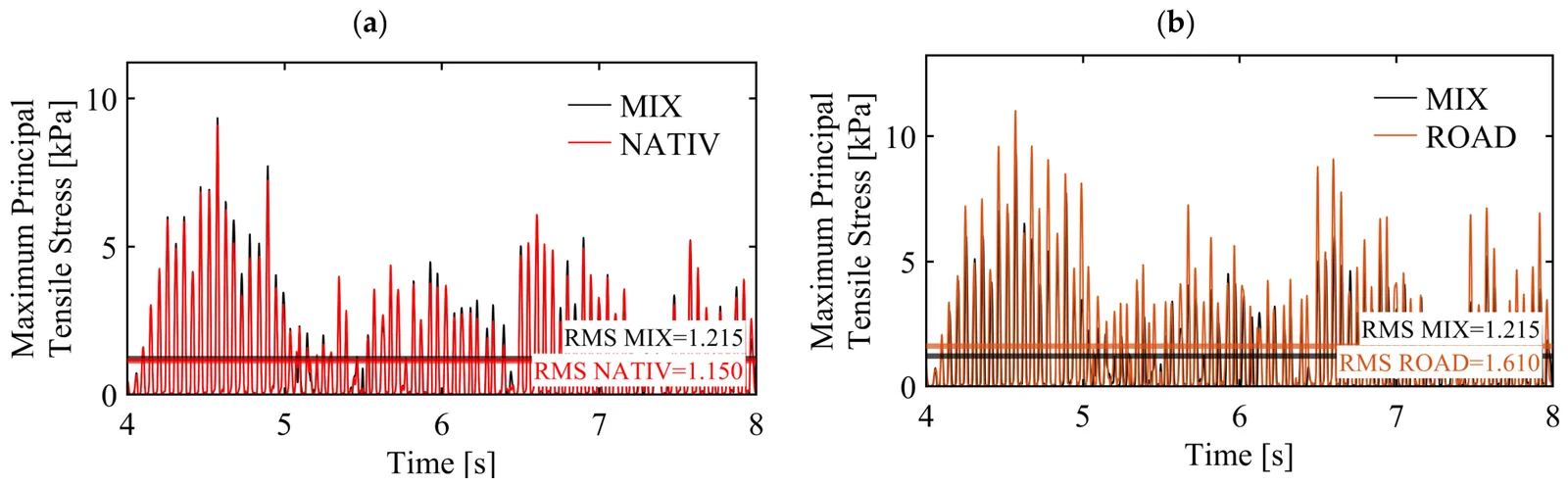
Comparison of maximum principal tensile stress at the middle of the main span (P2–P3): (**a**) MIX vs. NATIV, (**b**) MIX vs. ROAD.

**Figure 22:**
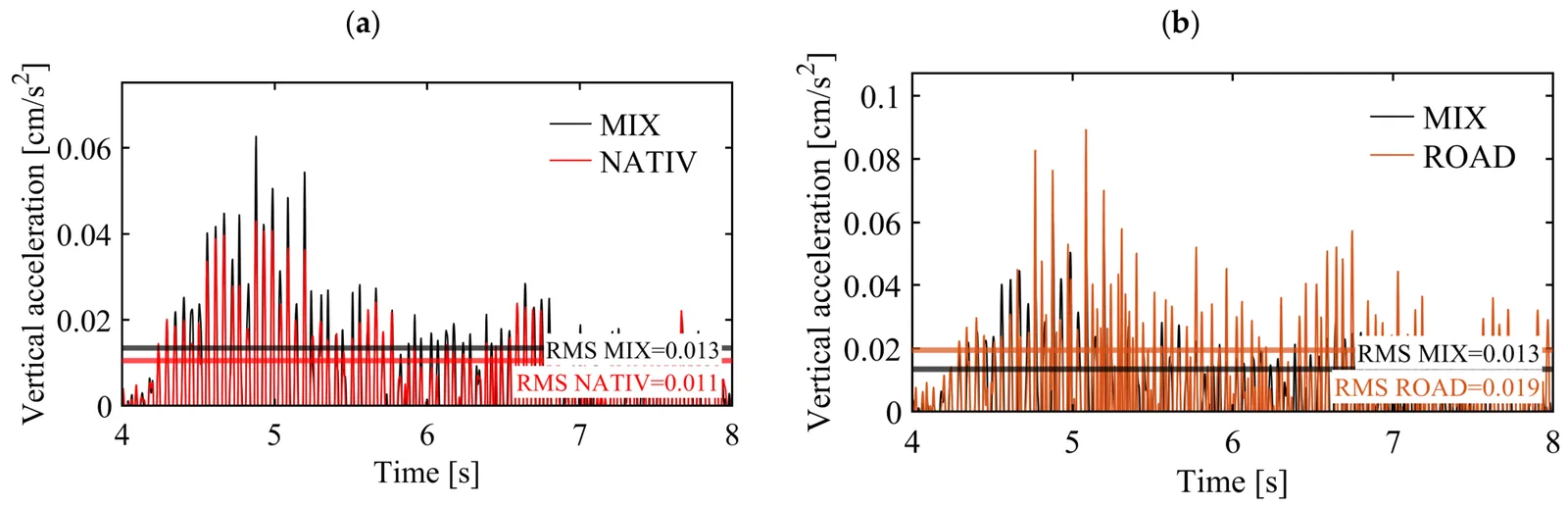
Comparison of vertical accelerations at the middle of the side span (P3–P4): (**a**) MIX vs. NATIV, (**b**) MIX vs. ROAD.

**Table 1:** Excitation sweep–target frequency combinations used in the wave-propagation and ODS analyses.

Target Frequency	Sweep Range	Sweep ID
20 Hz	8–50 Hz	SW1_8–50 Hz
	8–50 Hz	SW2_8–50 Hz
	14–28 Hz	SW3_14–28 Hz
30 Hz	8–50 Hz	SW1_8–50 Hz
	8–50 Hz	SW2_8–50 Hz
	27–38 Hz	SW4_27–38 Hz
35 Hz	8–50 Hz	SW1_8–50 Hz
	8–50 Hz	SW2_8–50 Hz
	27–38 Hz	SW4_27–38 Hz

**Table 2:** Propagation velocities identified from cross-correlation of the measured support signals.

f [Hz]	Sweep	$v_{12}$ [m/s]	$v_{13}$ [m/s]	$v_{23}$ [m/s]	$v_{23}$ Check [m/s]
20	SW1_8–50 Hz	135	311	500	497
20	SW2_8–50 Hz	134	310	500	498
20	SW3_14–28 Hz	135	311	496	497
30	SW1_8–50 Hz	178	366	526	525
30	SW2_8–50 Hz	140	296	434	435
30	SW4_27–38 Hz	141	365	672	672
35	SW1_8–50 Hz	151	310	444	444
35	SW2_8–50 Hz	150	338	530	528
35	SW4_27–38 Hz	151	311	444	445

**Table 3:** Quantitative experimental–theoretical ODS phase agreement for all analyzed sweep–frequency combinations.

f [Hz]	Sweep	$e\varphi_{2}$ [°]	$e\varphi_{3}$ [°]	$e\varphi_{4}$ [°]	$RMSE_{\varphi }$ [°]	$RMSE_{\varphi ,\%}$ [%]
20	SW1_8–50 Hz	1.2	2.0	6.2	3.82	2.12
20	SW2_8–50 Hz	1.1	0.3	6.5	3.81	2.12
20	SW3_14–28 Hz	3.1	0.4	4.4	3.11	1.73
30	SW1_8–50 Hz	1.6	0.2	1.9	1.44	0.80
30	SW2_8–50 Hz	0.6	5.2	1.3	3.11	1.73
30	SW4_27–38 Hz	4.3	5.2	4.9	4.81	2.67
35	SW1_8–50 Hz	5.0	5.4	6.7	5.74	3.19
35	SW2_8–50 Hz	3.5	2.6	1.8	2.72	1.51
35	SW4_27–38 Hz	3.8	0.2	2.9	2.76	1.53

**Table 4:** Representative propagation velocities adopted for the frequency-independent engineering application, together with the ranges identified from the individual excitation sweeps.

Propagation Section	Supporting-Medium Condition	Apparent Propagation Velocity [m/s]	Range of Identified Velocities [m/s]
$P_{1}\text{–}P_{2}$	Native subsoil	$v_{NATIV}$ = 146	134–178
$P_{2}\text{–}P_{3}$	Road substructure	$v_{ROAD}$ = 505	434–672

**Table 5:** Representative material absorption coefficients adopted for the frequency-independent engineering application.

Propagation Section	Supporting-Medium Condition	Material Absorption Coefficient [$m^{-1}$]	Range of Identified Coefficients [$m^{-1}$]
$P_{1}\text{–}P_{2}$	Native subsoil	$\alpha_{NATIV}$ = 0.05788	0.04933–0.06821
$P_{2}\text{–}P_{3}$	Road substructure	$\alpha_{ROAD}$ = 0.002984	0.000235–0.004719

**Table 6:** Supporting-medium configurations adopted in the three propagation scenarios.

Scenario	Supporting-Medium Type in Section		
	A1–P1–P2	P2–P3	P3–P4–A2
NATIV	Native subsoil	Native subsoil	Native subsoil
ROAD	Road substructure	Road substructure	Road substructure
MIX	Native subsoil	Road substructure	Native subsoil

**Table 7:** RMS and peak values of maximum principal tensile stress at nine control locations for the NATIV, ROAD, and MIX scenarios, with percentage deviations relative to MIX.

Control Location	NATIV		ROAD		MIX		NATIV vs. MIX		ROAD vs. MIX	
	RMS [kPa]	Peak [kPa]	RMS [kPa]	Peak [kPa]	RMS [kPa]	Peak [kPa]	RMS [%]	Peak [%]	RMS [%]	Peak [%]
A1-P1	6.79	36.92	6.81	38.94	6.81	37.89	−0.29	−2.56	0.00	+2.77
P1	11.18	57.50	11.53	65.10	11.20	64.90	−0.18	−11.40	+2.95	+0.31
P1-P2	6.52	39.70	6.60	42.70	6.54	40.10	−0.31	−1.00	+0.92	+6.48
P2	3.96	23.90	4.04	29.70	3.96	24.10	0.00	−0.83	+2.02	+23.24
P2-P3	1.15	9.08	1.61	11.20	1.22	9.35	−5.74	−2.89	+31.97	+19.79
P3	1.66	9.14	2.36	13.44	1.98	10.86	−16.16	−15.84	+19.19	+23.76
P3-P4	1.11	9.77	1.67	12.52	1.37	11.29	−18.98	−13.46	+21.90	+10.89
P4	1.59	10.40	2.40	16.66	1.82	12.20	−12.64	−14.75	+31.87	+36.56
P4-A2	0.97	5.21	1.14	6.37	1.04	5.31	−6.73	−1.88	+9.62	+19.96

## Data Availability

The original contributions presented in this study are included in the article. Further inquiries can be directed to the corresponding author.
